# Widespread Protein Aggregation as an Inherent Part of Aging in *C. elegans*

**DOI:** 10.1371/journal.pbio.1000450

**Published:** 2010-08-10

**Authors:** Della C. David, Noah Ollikainen, Jonathan C. Trinidad, Michael P. Cary, Alma L. Burlingame, Cynthia Kenyon

**Affiliations:** 1 Department of Biochemistry and Biophysics, University of California San Francisco, San Francisco, California, United States of America; 2 Graduate Program in Biological and Medical Informatics, University of California San Francisco, San Francisco, California, United States of America; 3 Mass Spectrometry Facility, Department of Pharmaceutical Chemistry, University of California San Francisco, San Francisco, California, United States of America; University of Cambridge, United Kingdom

## Abstract

Aberrant protein aggregation is a hallmark of many age-related diseases, yet little is known about whether proteins aggregate with age in a non-disease setting. Using a systematic proteomics approach, we identified several hundred proteins that become more insoluble with age in the multicellular organism *Caenorhabditis elegans*. These proteins are predicted to be significantly enriched in β-sheets, which promote disease protein aggregation. Strikingly, these insoluble proteins are highly over-represented in aggregates found in human neurodegeneration. We examined several of these proteins in vivo and confirmed their propensity to aggregate with age. Different proteins aggregated in different tissues and cellular compartments. Protein insolubility and aggregation were significantly delayed or even halted by reduced insulin/IGF-1-signaling, which also slows aging. We found a significant overlap between proteins that become insoluble and proteins that influence lifespan and/or polyglutamine-repeat aggregation. Moreover, overexpressing one aggregating protein enhanced polyglutamine-repeat pathology. Together our findings indicate that widespread protein insolubility and aggregation is an inherent part of aging and that it may influence both lifespan and neurodegenerative disease.

## Abstract 2

Several hundred proteins become insoluble and aggregation-prone as a consequence of aging in *Caenorhabditis elegans*. The data indicate that these proteins influence disease-related protein aggregation and toxicity.

## Author Summary

In neurodegenerative diseases, such as Alzheimer's disease and Huntington's disease, specific proteins escape the cell's quality-control system and associate together, forming insoluble aggregates. Until now, little was known about whether proteins aggregate in a non-disease context. In this study, we discovered that the aging process itself, in the absence of disease, leads to the insolubilization and increased aggregation propensity of several hundred proteins in the roundworm *Caenorhabditis elegans*. These aggregation-prone proteins have distinct structural and functional proprieties. We asked if this inherent age-dependent protein aggregation impacts neurodegenerative diseases. We found that proteins similar to those aggregating in old worms have also been identified as minor components of human disease aggregates. In addition, we showed that higher levels of inherent protein aggregation aggravated toxicity in a *C. elegans* Huntington's disease model. Inherent protein aggregation is a new biomarker of aging. Understanding how to modulate it will lead to important insights into the mechanisms that underlie aging and protein aggregation diseases.

## Introduction

The regulation of protein homeostasis (proteostasis) plays an essential role in preventing protein aggregation. As organisms age, the tightly regulated balance of gene expression levels, quality control, and protein disposal is disrupted. For example, cellular systems responsible for protein degradation become less efficient with age [1],[2]. In addition, chaperone levels change in older animals [3]. Aging is also associated with increased oxidative stress, leading to irreversible oxidation and nitration of proteins, which impairs their degradation [4],[5]. These age-dependent changes in proteostasis are thought to facilitate the aberrant aggregation of specific proteins in the context of neurodegeneration and amyloidoses [6]. However, it is not clear to what extent this altered cellular environment also leads to protein aggregation during normal aging, in a non-disease context [7].

Although protein aggregation has mainly been associated with disease, a wide variety of proteins have the capacity to aggregate under extreme conditions in vitro [8]. Recent evidence suggests that partial unfolding of globular proteins can occur under physiological conditions and is sufficient to lead to protein aggregation [9]. Furthermore, almost all proteins contain buried self-complementary sequences that could promote the assembly of identical proteins into aggregates if exposed at the protein surface [10]. The aggregation of recombinant proteins is commonly observed in bacteria [11], and these inclusion bodies consist at least partly of amyloid-like structures [12]. Soluble proteins are found to aggregate in both *S. cerevisiae* and mammalian cells when these cells are challenged by inhibition of the proteasome [13],[14]. The eukaryotic cell has a built-in mechanism to deal with misfolded, aggregation-prone proteins, which becomes activated when the protein disposal system becomes impaired or overwhelmed. This mechanism involves the formation of the aggresome, an inclusion body located at the microtubule-organizing center that actively sequesters insoluble proteins [15].

The fact that proteostasis mechanisms decline with age suggests that normal cellular proteins might become more prone to aggregation. Furthermore, various proteins have been found to co-aggregate, albeit at low levels, together with major disease-aggregating proteins during age-dependent neurodegeneration [16],[17],[18]. However, a systematic evaluation of inherent protein aggregation during normal aging has been lacking [7]. In this study, we used a global proteomics approach to investigate the extent of age-dependent protein insolubility, a hallmark of protein aggregation, in wild-type *C. elegans*. We identified several hundred proteins that became more insoluble with age, and as predicted, almost all of the proteins we tested in vivo formed aggregates. We found that inhibiting the insulin/IGF-1 system, which slows aging, decreased the rate and extent of inherent protein insolubility and aggregation. Intriguingly, a significantly large fraction of RNAi clones that increase lifespan are predicted to target mRNAs encoding aggregation-prone proteins, raising the possibility that decreasing aggregation levels could prolong lifespan.

As misfolded proteins could potentially enhance disease-protein aggregation by disrupting proteostasis [19], we also asked whether age-dependent inherent protein aggregation would modify the course of aberrant disease-related protein aggregation. We found that increased expression of an aggregation-prone protein aggravated the paralysis caused by polyglutamine repeat aggregation, in spite of the fact that the two proteins did not co-aggregate. Moreover, a large fraction of proteins previously identified in a genome-wide screen for factors that influence polyglutamine repeat aggregation [20] consisted of proteins that we identified as insoluble. Especially strikingly was our finding that homologs of 31%–54% of the proteins previously identified in human disease aggregates were identified in our study. These findings reveal, for the first time, the large extent and the nature of age-dependent protein insolubility and aggregation in a non-disease context. Our results suggest that inherent protein aggregation has the potential to influence lifespan and protein aggregation disease.

## Results

### Proteomic Identification of Detergent-Insoluble Proteins in *C. elegans*

**Figure 1:**
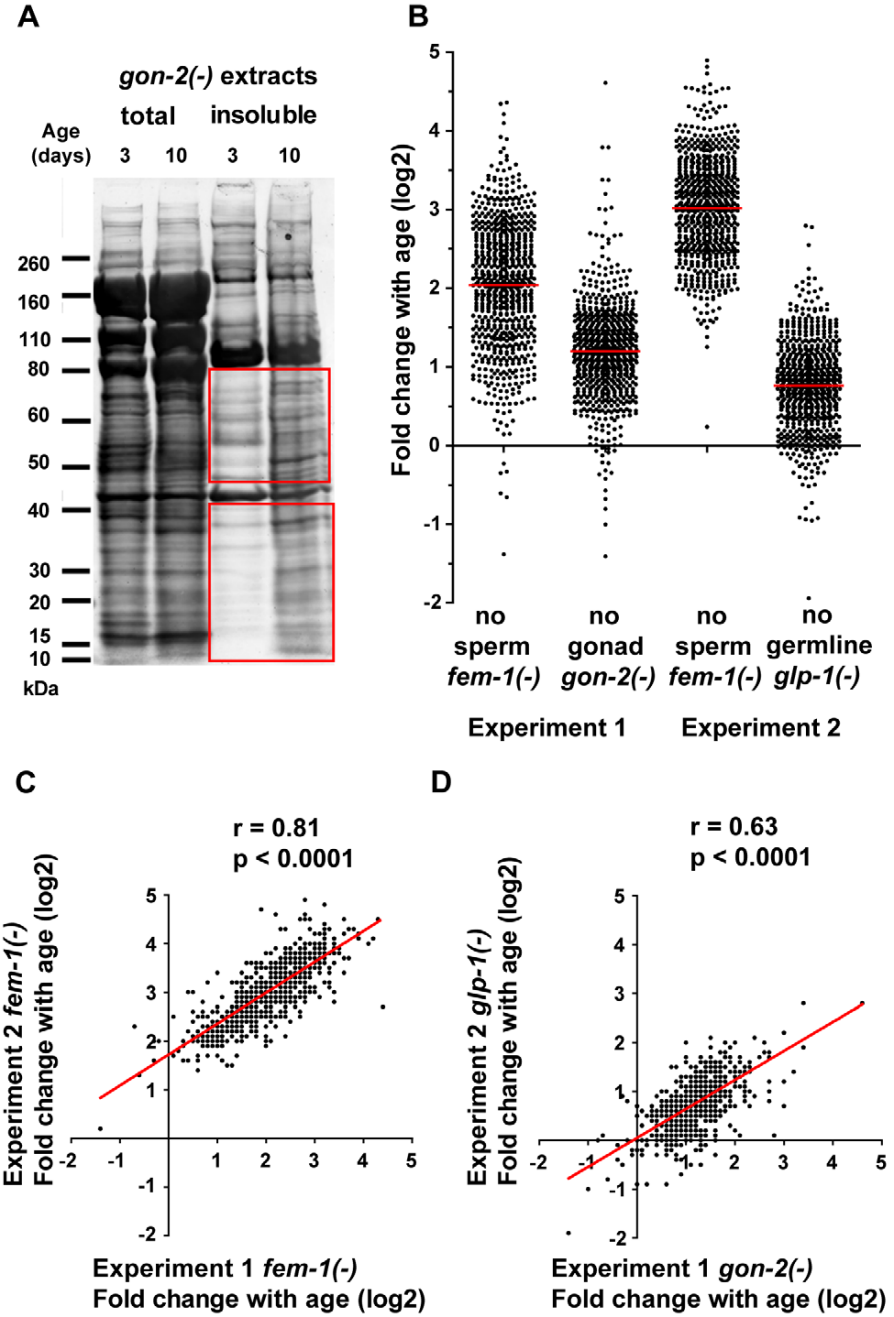
The majority of insoluble-prone proteins are consistently more insoluble with age in *C. elegans*. (A) Total and detergent-insoluble protein staining of *gon-2(−)*/gonad-less extracts using Sypro Ruby. Total protein fraction was diluted 1∶3 compared to the insoluble fraction. Quantification of insoluble proteins in the red outlined areas revealed a fold change of 3.5±0.8 (SD) on average between young and aged animals (two biological and two experimental repeats). (B) Distribution of the fold change shown in logarithm base 2 in levels of insoluble proteins identified in aged animals (when half the population is alive) compared to young animals (Day 3 of adulthood at 25°C). Each dot represents the fold change for one insoluble-prone protein. Red bar indicates the mean. The fold changes of proteins that were identified in both independent experiments are shown for each strain in Experiment 1 and Experiment 2. These results show that proteins identified in the formic acid soluble fraction have a tendency to accumulate with age. 711 proteins were present in all four samples (defined as having an iTRAQ peak above 25 counts in young and/or in old animals) (Experiment 1 and Experiment 2). Of these, some were excluded from the fold change calculations because their iTRAQ peak was too low (≤25 counts) in young animals. Fold changes were calculated in Experiment 1 for 698 of 711 insoluble proteins in *fem-1(−)* animals and 670 of 711 insoluble proteins in *gon-2(−)* animals and in Experiment 2 for 692 of 711 insoluble proteins in *fem-1(−)* and 695 of 711 insoluble proteins in *glp-1(−)*. (C–D) Changes in insolubility with age in the two independent biological replicates were strongly correlated. Age-dependent insolubility fold changes are plotted for both Experiments 1 and 2, comparing *fem-1(−)* animals (C) and comparing *gon-2(−)* to *glp-1(−)* animals (D). Spearman *r* correlation and two-tailed *p* values were calculated for each set: *fem-(−)*: *r* = 0.81, *p*<0.0001; *gon-2(−)*/*glp-1(−)*: *r* = 0.63, *p*<0.0001.

Proteins known to aggregate in disease, such as tau protein and amyloid-β, remain insoluble in strong-detergent buffers [21],[22],[23] but can be solubilized by formic acid or urea and analyzed on SDS-PAGE gels. We used similar conditions to look for proteins that might be prone to aggregate during the normal aging process. We observed that a significant fraction of the *C. elegans* proteome remained insoluble in a strong-detergent buffer (Figure 1A). Many of these insoluble proteins were present at a higher level in old animals, suggesting that aging potentiates the tendency of proteins to become insoluble. Specifically, the mean fold change of proteins that became more insoluble with age in sterile [gonad-less *gon-2(−)*] animals was ∼3.5±0.8 (SD). The patterns of bands on the SDS-PAGE gel in the total and insoluble fractions were not the same, indicating that the propensity to become insoluble with age does not affect all proteins equally. Some insoluble protein bands were present at similar levels in young and aged animals, suggesting these include proteins that are not aggregation-prone but rather proteins such as tubulin and cuticular collagen proteins that are functional in an insoluble state. 

To determine the identity of the insoluble proteins, we collected young and old animals and adapted a stringent sequential protein solubilization protocol established previously to extract disease-aggregated proteins. Briefly, following removal of dead worms by sucrose separation, we repeatedly removed cytosolic-soluble proteins in high-salt buffer and membrane proteins in a detergent buffer containing both strong anionic and nonionic detergents (Figure S1A). The remaining, insoluble, proteins were solubilized with formic acid. Some proteins, such as those associated with the worm cuticle, remained in the pellet. This acid soluble fraction was digested with trypsin, and multidimensional LC-MS/MS was used to identify 1,125 and 856 proteins in two biological replicates. Sixty-four percent of the proteins identified in the insoluble fraction in the first biological experiment were also found in the second experiment, and conversely 85% of the proteins identified in the second experiment were identified in the first experiment (Figure S1B). This overlap between biological replicates is comparable to previously published proteomic data [24],[25],[26]. For comparison, if we sampled randomly 1,125 and 856 proteins in the *C. elegans* proteome (consisting of 7,826 proteins previously detected by mass spectrometry), we would expect to detect at most 123 proteins in both samples (11% and 14%, respectively) by chance. The cumulative hypergeometric probability of detecting 725 or more proteins in both experiments is less than 1E-100. For further analysis, we focused on 711 proteins that were identified in both experiments and passed a stringent set of mass spectrometry related quality-control criteria (see Methods) (Table S1).

### Protein Insolubility Increases with Age in *C. elegans*

To quantify changes with age in both of the two biological replicates described above, we compared the levels of insoluble proteins extracted from young adult *C. elegans* (Day 3 of adulthood) to those extracted from an aging population (determined as the time point when half of the population remained alive). To obtain a large synchronized population of aged animals, we used temperature-induced sterility mutants (described below), which were maintained at 25°C. The extent of age-dependent insolubility in these strains was quantified by conducting quantitative mass spectrometry using the stable-isotope iTRAQ reagents [27], which allowed us to analyze four different samples simultaneously (Figure S2). In each of the two experiments, we analyzed *fem-1(−)* mutants, which are defective in sperm production [28]. We found that 691 proteins (Experiment 1) and 710 proteins (Experiment 2) out of the 711 insoluble proteins accumulated by 1.5-fold or more with age. In Figure 1B, we used a logarithm base 2 scale to represent the wide distribution of age-dependent changes in the levels of insoluble forms of the different insoluble-prone proteins. Because *fem-1(−)* mutants accumulate unfertilized oocytes, as do wild-type animals to a lesser extent, we hypothesized that the extent of insolubility could be explained partially by decreased oocyte quality with age [29]. To control for this, we also analyzed changes in insolubility with age in mutant strains lacking oocytes (Figure 1B). For added stringency, we chose to analyze two different strains as biological replicates: the gonad-less mutant *gon-2(−)*, and the germline-deficient mutant *glp-1(−)*. In these two strains without oocytes, we found that 621 proteins (Experiment 1) and 486 proteins (Experiment 2) out of the 711 insoluble proteins present in both samples accumulated by 1.5-fold or more with age. Conversely, the levels of only two insoluble proteins (CSQ-1 and PAT-10) decreased by over 1.5-fold with age in both strains without oocytes. Combining the quantification of both experiments (examining insoluble proteins from animals with and without oocytes), we have found a set of 461 proteins that consistently become 1.5-fold or more insoluble with age (Table S1). We will refer to these 461 proteins as the “age-dependent insoluble” set. Overall, these results show that the aging process is associated with a large increase in protein insolubility.

To ask whether individual proteins behave in a reproducible fashion between experiments, for each protein, we plotted the fold change in insolubility with age for both biological repeats (Figure 1C and 1D). We found a highly significant correlation between fold changes in insolubility measured by iTRAQ in both replicate analyses of *fem-1(−)* animals (*r* = 0.8, *p*<0.0001). The correlation between fold changes in insolubility in *gon-2(−)* and *glp-1(−)* animals was slightly lower but still very significant (*r* = 0.6, *p*<0.0001). This reproducibility indicates that age-dependent protein insolubility is not a stochastic process, but rather that certain proteins have properties that make them more prone to insolubility with age.

In somatic tissues (assayed using either *gon-2(−)* or *glp-1(−)* animals), we observed a group of 250 insoluble-prone proteins that were not more insoluble with age, which we will refer to as the “age-independent insoluble” set. Among these, 60 insoluble-prone proteins remained at similar levels with age in both strains. This group contains the majority of the cytoskeleton proteins identified (with the notable exception of intermediate filaments and lamin-1) (Table S2). Some cytoskeletal proteins such as gamma-tubulin have been shown to be present in an insoluble but functional state in the cell [30]. We note that under specific conditions, tubulin can also assemble irreversibly into non-specific polymers (i.e. aggregates) [31]. Interestingly, intermediate filaments form cages delimiting the aggresome [32] and therefore their accumulation with age may represent an effort to segregate age-related aggregating proteins. By extrapolation, other proteins present in our insoluble set may be functional and yet insoluble. Conversely, we predict that misfolded proteins that assemble together to form aggregates will also be found in our insoluble fraction, as shown for disease aggregated proteins. The tendency of ∼2/3 of the insoluble proteins identified to become more insoluble with age in all strains examined strongly suggests that protein insolubility is facilitated by the aging process. In experiments described below, we found that, when fluorescently tagged, all but one (very small) protein we examined formed insoluble aggregates in the animal. Therefore, we will refer to these insoluble proteins provisionally as aggregation-prone proteins.

Overall, we have identified a reproducible set of several hundred insoluble proteins predicted to include aggregation-prone proteins that become more insoluble with age.

### Age-Dependent Aggregation Occurs in Different Tissues

To better understand inherent protein aggregation in vivo, we selected two proteins, RHO-1 and KIN-19, for more detailed analysis. These proteins became 2-fold or more insoluble with age in all the strains as quantified by mass spectrometry. Both proteins are highly conserved in mammals: their mammalian homologs are transforming protein RhoA and casein kinase 1 isoform alpha (CK1α). Interestingly, CK1α is found tightly associated with pathological intracellular inclusions in Alzheimer's disease that mainly contain tau protein, as well as in sporadic inclusion body myositis (sIBM) [33],[34].

**Figure 2:**
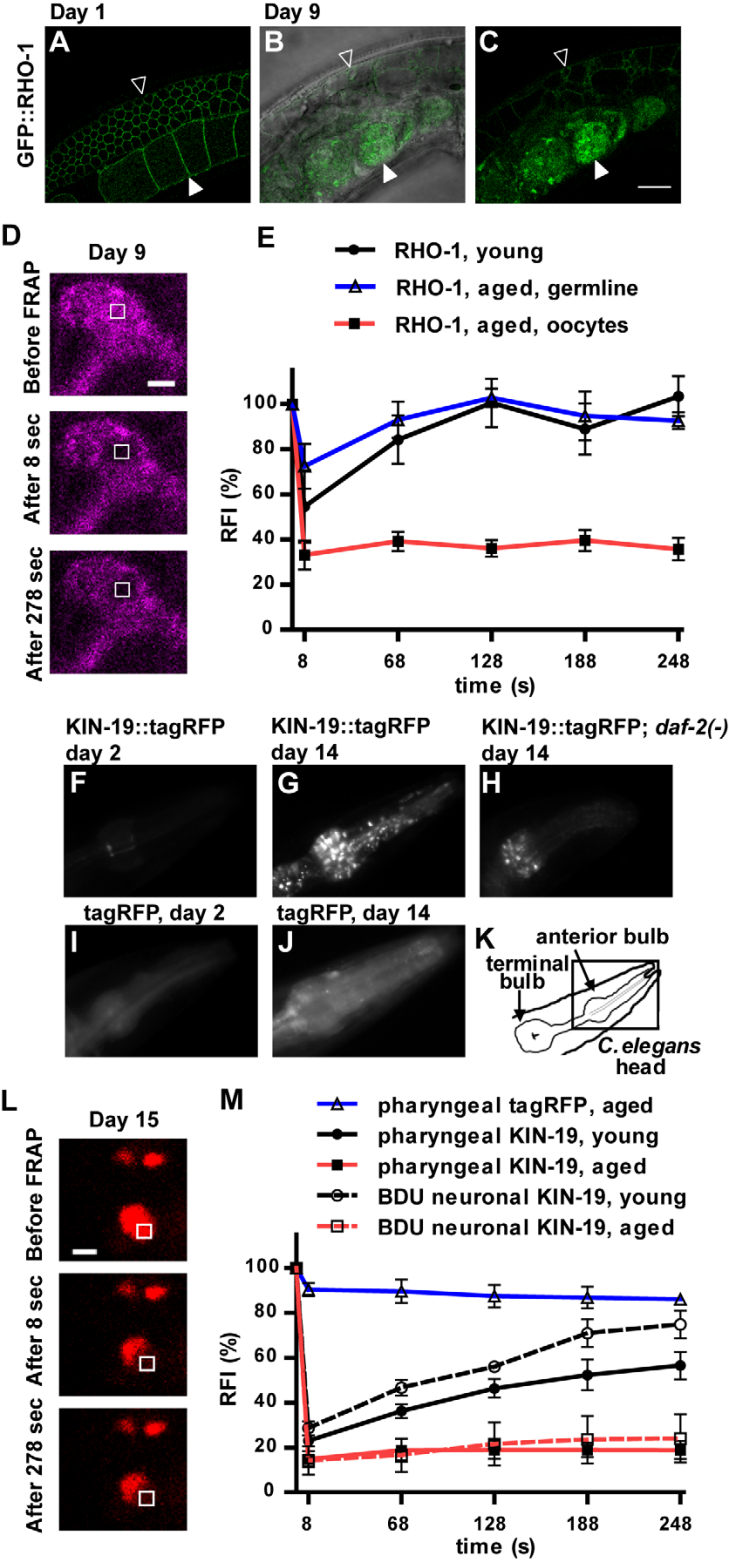
Age-dependent protein aggregation can occur in different tissues. (A–B) *Ppie-1::gfp::rho-1*-expressing animal, Day 1 (A) and Day 9 (B–C). In our proteomic analysis, we identified RHO-1 as a protein prone to aggregate with age both in the reproductive and somatic tissues. GFP::RHO-1, expressed in the germline, was localized to germline stem cell (open arrowhead) and oocyte (full arrowhead) membranes in young animals. With age, GFP::RHO-1 also accumulated in sclerotic oocytes in the uterus (full arrowhead). Scale bar: 20 µm. (D) FRAP-immobile GFP::RHO-1 in aged oocytes. GFP::RHO-1 is pseudocolored in magenta. Laser setting: 25% in 0.85 µm^2^ (white open box). Scale bar: 2 µm. (E) Quantification of relative fluorescence intensity (RFI) during recovery. We found no recovery of GFP::RHO-1 in sclerotic oocytes in aged animals (Day 12, *N* animals = 5, *N* puncta evaluated = 5) but rapid fluorescence recovery for GFP::RHO-1 localized to the germ-line cell membrane in young animals (t_1/2_ = 35 s, Day 1, *N* animals = 5, *N* puncta evaluated = 5) and in aged animals (t_1/2_ = 17 s, Day 12, *N* animals = 3, *N* puncta evaluated = 4). (F–K) Formation of KIN-19::tagRFP puncta in the anterior pharyngeal bulb (metacorpus) with age. *Pkin-19::kin-19::tagrfp* animals (F and G); *daf-2(e1370)*; *Pkin-19::kin-19::tagrfp* animals (H); control *Pkin-19::tagrfp* animals (I and J). (F–J) 10 ms exposure, 100×. (K) Schematic of *C. elegans*' pharynx, boxed area is shown in F–J. (L) FRAP-immobile KIN-19::tagRFP puncta in the anterior pharyngeal bulb of 15-day-old *Pkin-19::kin-19::tagrfp* animal. Laser settings: 40% in 0.46 µm^2^ (white open box). Scale bar: 1 µm. (M) We found no fluorescence recovery of pharyngeal or BDU neuronal KIN-19::tagRFP in aged animals (Day 12, *N* animals = 5, *N* puncta evaluated = 5 and Day 12 and 14, *N* animals = 5, *N* puncta evaluated = 5, respectively). KIN-19::tagRFP was able to diffuse back at a slow rate in young animals both in the pharynx and neurons (t_1/2_ = 107 s, Day 1, *N* animals = 4, *N* puncta evaluated = 5 and t_1/2_ = 151 s, Day 3, *N* animals = 5, *N* puncta evaluated = 5, respectively). The few puncta formed by tagRFP alone contained highly mobile protein in aged animals (Day 12; *N* animals = 5, *N* puncta evaluated = 5).

Our proteomic analysis identified a considerable increase in insolubility with age in strains that contained oocytes. To test whether aggregation occurs in the reproductive tissue, we examined the aggregation-prone protein RHO-1 tagged with GFP under the control of the germline-specific *pie-1* promoter (Figure 2A) [35]. In disease models for aberrant protein aggregation, protein aggregation is characterized by the assembly of the misfolded protein into microscopically visible aggregates [36]. We found that GFP::RHO-1, localized to the oocyte membranes in young adults, accumulated with age in dense patches in the sclerotic oocytes of older animals (Day 9) (Figure 2B and 2C). Another hallmark of protein aggregation is the transition from a soluble to an insoluble state, resulting in reduced mobility of the aggregated protein. Fluorescent recovery after photobleaching (FRAP) is a standard method for visualizing this transition to a state of aggregation [37],[38]. FRAP analysis indicated that GFP::RHO-1 became immobile in these sclerotic structures, consistent with its aggregating with age (Figure 2D and 2E). In contrast, GFP::RHO-1 remained mobile in the membranes of the germline stem cells both in young and aged animals (respectively, t_1/2_ = 35 s and t_1/2_ = 17 s). Thus, RHO-1 aggregation is restricted to the oocytes. This finding supports the idea that oocyte-specific aggregation could explain the difference between the relatively high levels of age-dependent insolubility in *fem-1(−)* animals (which contain oocytes) versus the lower levels present in *glp-1(−)* and *gon-2(−)* animals (which do not). Thus, degenerating oocytes in wild-type animals may create a favorable environment for protein aggregation. Consistent with this interpretation, as in wild type, the oocytes acquired a sclerotic appearance in old *fem-1(−)* animals, when viewed with Normarski optics.

**Figure 3:**
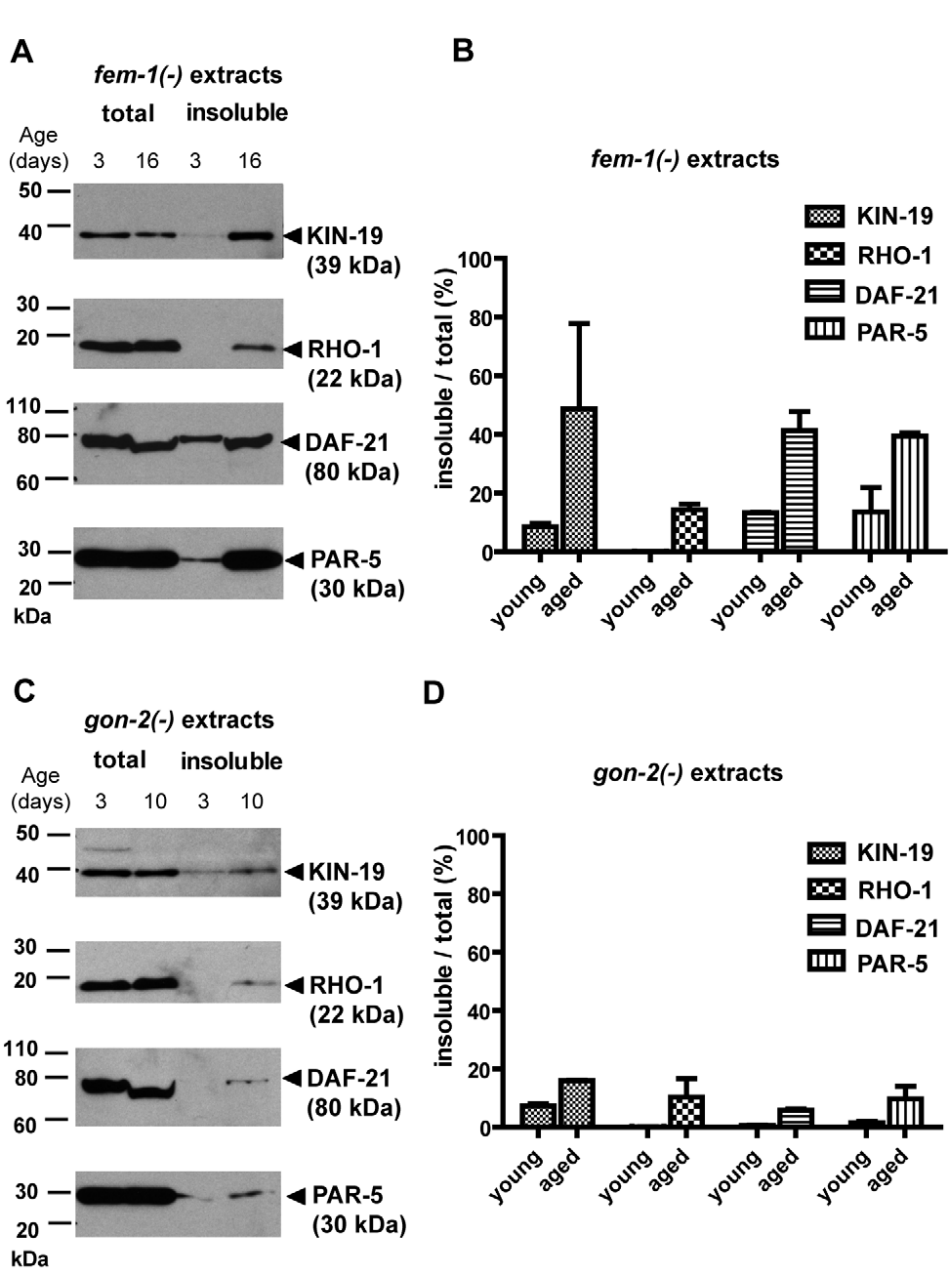
Insoluble but not total levels of four aggregation-prone proteins increased with age. (A and C) Western blot detection of KIN-19 (CK1α), RHO-1, DAF-21 (Hsp90), and PAR-5 (14-3-3) in young and aged animals containing either somatic and germline tissue [*fem-1(−)*, (A)] or containing only somatic tissues [*gon-2(−)*, (C)]. The total fraction (Urea and SDS buffer) contains all proteins and the detergent-insoluble fraction contains aggregation-prone proteins. The total protein fraction was diluted 1∶3 compared to insoluble fraction. Arrowheads mark the protein bands corresponding to the aggregation-prone candidates. Overall, Western blot analysis confirms our mass spectrometry results demonstrating a large increase in insolubility with age. With age, we noted a slight decrease in the size of full-length DAF-21 (less than 10 kDa). (B and D) Quantification of the fractional increase in aggregated levels compared to total levels of each candidate evaluated by Western blot. These results demonstrate that age-dependent insolubility for each of the four proteins we examined occurs independently of an increase in total protein levels. Extracts from two biologically independent experiments were evaluated. Error bars indicate SEM.

To evaluate somatic tissue aggregation, we expressed KIN-19 tagged with the recently developed monomeric fluorescent protein “tagRFP” using its endogenous promoter [39]. We found that this fusion protein was most highly expressed in the pharynx and in a pair of neuronal processes identified by their position as BDU neurons (Figure S3A). We determined that the level of transgenic KIN-19::tagRFP in the whole animal was similar to that of endogenous KIN-19, as measured by Western blot (Figure S3B). Transgenic animals showed no notable defects and had a lifespan similar to that of wild-type controls (Figure S3C). With age, we observed the formation of fluorescent-KIN-19 puncta throughout the anterior pharyngeal bulb, indicative of KIN-19 aggregation (schematized in Figure 2K) (Figure 2F and 2G). KIN-19::tagRFP puncta were found throughout the cytoplasm in pharyngeal muscle and marginal cells. We evaluated KIN-19::tagRFP puncta formation in the pharynx at different time points throughout life in a population of animals (Figure S3D). A small proportion of the population started to accumulate puncta at Day 3 of adulthood, implying a critical threshold for aggregation at this time point. Thereafter, the number of animals displaying pharyngeal KIN-19 puncta increased rapidly until Day 9, when over 80% of the population contained puncta. To test whether the proteins within the KIN-19::tagRFP puncta were immobile, we measured FRAP in defined areas of individual punctae. Even after 270 s, we observed no recovery in fluorescence in the majority of puncta examined, demonstrating that KIN-19::tagRFP could not diffuse back into the bleached area and consistent with a state of aggregation (Figure 2L and 2M). A small proportion of the KIN-19::tagRFP puncta were composed of mobile protein (Table 1). In contrast, control animals expressing only the tagRFP reporter formed only a few puncta with age (Figure 2I and 2J), and all these puncta contained mobile tagRFP as determined by FRAP (Figure 2M). We also performed FRAP on two KIN-19::tagRFP punctate-like structures in the base of the pharynx that were always present in Day 1 animals (Figure 2F). We observed partial recovery of KIN-19::tagRFP fluorescence after photobleaching with a recovery half-life of 107 s in young animals (Figure 2M). The relatively slow recovery rate of KIN-19::tagRFP compared to the rapid recovery rate of GFP::RHO-1 fluorescence in young animals suggests that a fraction of KIN-19::tagRFP is already immobile in these animals, consistent with our Western blot analysis (Figure 3).

**Table 1:** Pharyngeal fluorescent puncta in *daf-2(−)* mutants contain mobile KIN-19.

	Total KIN-19::tagRFP Puncta Evaluated	Number of Animals	Boxed Area Bleached(1)	Boxed Area Bleached(1)	Whole Puncta Bleached(2)
			Immobile (No recovery)	Mobile (Recovery)	Mobile (No recovery or partial recovery)
**wild-type**	36	16	25	5	6
***daf-2(e1370)***	34	18	6	6	22

The assay was done in a blind fashion in which the identity of the samples was concealed. FRAP was performed on KIN-19::tagRFP puncta in a wild-type background between Day 12–15 and in a *daf-2(e1370)* background between Day 13–15 of adulthood. Recovery after photobleaching was estimated between 2.5 to 4 min after bleaching.

(1) This category comprises puncta where the laser beam bleached the targeted boxed area without affecting the non-targeted area to the same extent. Puncta in (1) where we observed no recovery after photobleaching were determined to contain immobile KIN-19::tagRFP protein. Conversely, KIN-19::tagRFP protein was estimated to be mobile when we observed recovery of the area bleached.

(2) This category comprises puncta where the laser beam uniformly bleached both targeted and non-targeted parts of the puncta. We observed either no-recovery or a partial recovery in fluorescence of the whole puncta. These puncta were estimated to contain mobile KIN-19::tagRFP protein.

Mobile compared to immobile KIN-19::tagRFP puncta in *daf-2(+)* and *daf-2(−)* background: Fisher's exact test *p*<0.0001.

In addition, we measured KIN-19::tagRFP mobility in the BDU neurons, where it was relatively highly (and uniformly) expressed compared to other neurons. We did not observe the formation of distinct KIN-19::tagRFP puncta in these processes in young or in aged animals, but nevertheless FRAP analysis showed that KIN-19::tagRFP becomes immobile in older BDU neurons (Figures 2M and S4A). In young animals (Day 3), we found partial recovery after photobleaching at a rate similar to that observed in the young pharynx (t_1/2_ = 151 s, Figures 2M and S4B). The generally low expression levels of KIN-19::tagRFP in other neuronal processes prevented us from conducting sufficient FRAP analysis to determine whether their KIN-19::tagRFP was mobile or not.

Overall, our in vivo data confirm that proteins prone to insolubility with age that were identified by our proteomic analysis have the potential to aggregate in different tissues in an age-dependent manner in the animal.

### Age-Dependent Protein Aggregation Is Not Correlated with a Change in Total Protein Levels

The overall increase in aggregation propensity with age could be enhanced by a proportional increase in the total levels of these aggregation-prone proteins. To test this hypothesis, we chose four proteins for which antibodies were available, the *C. elegans* homologs of casein kinase I alpha, CK1α (KIN-19), Rho1, Hsp90 (DAF-21), and 14-3-3 (PAR-5), and asked whether their levels increased with age. Young and aged animals were solubilized either in Urea/SDS buffer to obtain total protein extracts or sequentially extracted in RIPA followed by Urea/SDS buffer to isolate insoluble proteins in the pellet. Quantification of total protein for each candidate showed that levels remained either constant or decreased slightly with age (Figure S5A). We observed the largest change for total DAF-21 levels, which were reduced on average by 1.47-fold with age. [The reduction in DAF-21 levels was correlated with a small shift in molecular weight and the appearance of a cleavage product in the total and RIPA-soluble fraction in the aged population (Figure 3 and S5C)]. Quantification of the insoluble levels relative to total levels of these candidates revealed a significant increase in insolubility with age in animals with and without reproductive tissues (Figure 3, Mann Whitney test, *fem-1(−)*, *p* = 0.003; *gon-2(−)*, *p* = 0.01). Our results demonstrate that, at least for these four proteins, increased protein levels are not the direct cause of age-dependent protein aggregation. Interestingly, our analysis also showed that only a fraction of the total amount of protein available will become insoluble in aged animals. Averaging our Western blot results for all four candidates examined, we found that 10.4% of the protein available in *gon-2(−)* animals and 35.9% of the protein available in *fem-(−)* animals is insoluble in aged animals (Figure S5B). Therefore, these results suggest that the majority of the pool of aggregation-prone proteins remains detergent-soluble.

**Figure 4:**
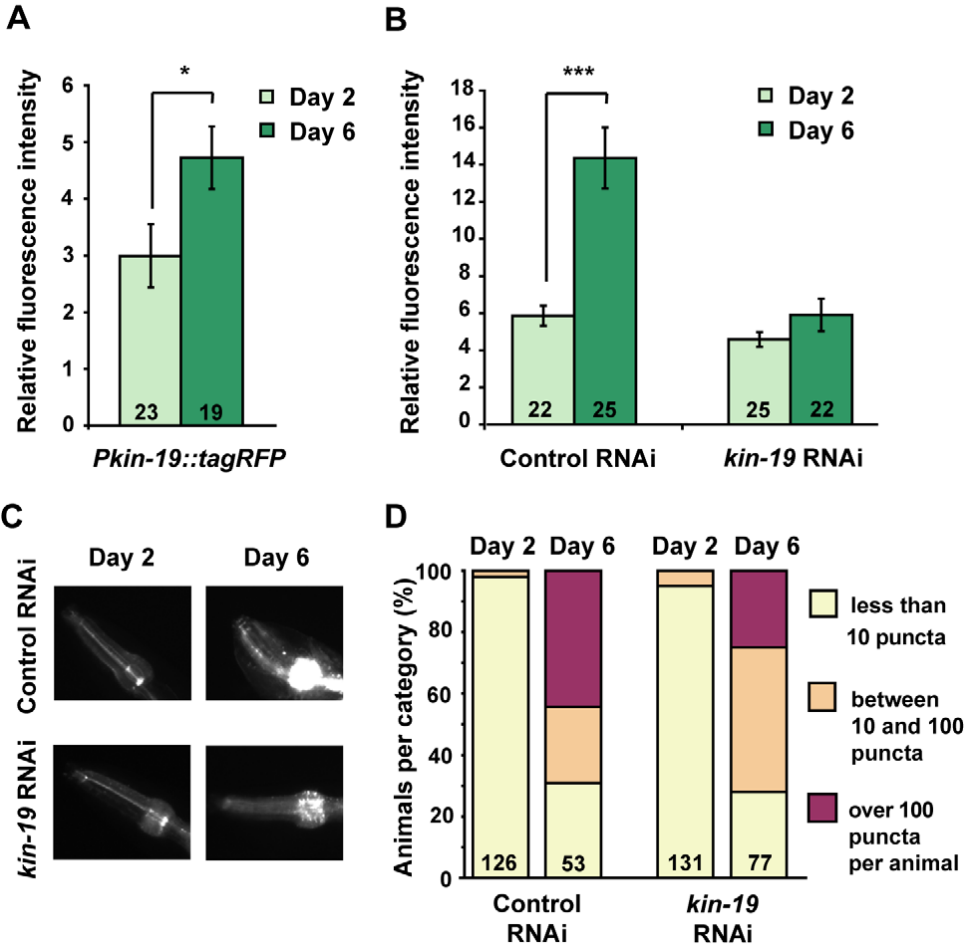
Reducing KIN-19::tagRFP levels does not prevent age-dependent protein aggregation in the pharynx. (A) The activity of the *kin-19* promoter was up-regulated with age. Fluorescence from the tagRFP reporter driven by the *kin-19* promoter increased by 1.6-fold between Day 2 and Day 6 in *Pkin-19::tagrfp* animals (Unpaired *t* test *p = 0.03). Relative fluorescence quantification in the anterior pharyngeal bulb is shown, 5 ms exposure. Numbers of animals quantified are given in the histogram bars. Error bars indicate SEM. (B) *kin-19* RNAi treatment prevented an increase in KIN-19::tagRFP levels with age (Day 2 versus Day 6 with *kin-19* RNAi, unpaired *t* test *p*>0.1). In comparison, KIN-19::tagRFP levels increased in the anterior pharyngeal bulb by 2.4-fold between Day 2 and Day 6 in *Pkin-19::kin-19::tagrfp* animals treated with control RNAi (Unpaired *t* test *** *p*<0.0001). Error bars indicate SEM. (C) Representative animals treated with *kin-19* RNAi or control RNAi. (D) Reducing KIN-19::tagRFP levels did not prevent its age-dependent aggregation. Animals were classified into three groups depending on the number of KIN-19::tagRFP puncta present in their anterior pharyngeal bulbs. For statistical analysis, we grouped both categories with more than 10 puncta and compared them to the category with less than 10 puncta. At Day 6, animals treated with control or *kin-19* RNAi had significantly more KIN-19::tagRFP aggregation than did control or *kin-19(RNAi)* animals on Day 2 (with *kin-19* RNAi, Day 2 and Day 6, Yates' Chi-square test: *p*<0.0001; with control RNAi, Day 2 and Day 6, Yates' Chi-square test: *p*<0.0001). Numbers of animals evaluated are shown in the histogram bars.

Although we did not observe higher total KIN-19 protein levels in the whole animal with age, we found a 1.6-fold up-regulation of *kin-19* promoter-directed expression in the pharynx, as measured by quantification of the transcriptional *Pkin-19::*tagRFP fluorescent reporter (Figure 4A). To exclude the hypothesis that KIN-19 aggregation in the pharynx was caused solely by an increase in the level of KIN-19, we reduced KIN-19::tagRFP levels using RNAi (Figure 4B) and asked whether the protein would still aggregate with age. Adult-only *kin-19*-RNAi treatment reduced the levels of KIN-19::tagRFP in 6-day-old adults to a level similar to that measured in 2-day-old controls. Under these conditions, we still observed significantly more KIN-19::tagRFP aggregation in older animals than in young animals (Figure 4C and 4D). We confirmed by FRAP that aggregates in 6-day-old *kin-19* RNAi-treated animals were formed by immobile KIN-19::tagRFP (Figure S4C). These findings show that preventing protein levels from increasing does not abrogate KIN-19 aggregation.

Overall, our data show that age-dependent protein aggregation is not necessarily caused by increased protein levels. Instead, other events associated with age can promote aggregation.

### Protein Insolubility in Young Animals

Whole-protein staining shows that many of the insoluble proteins detected in aged animals are also present in relatively young animals (Day 3 of adulthood at 25°C) at a time when no obvious signs of aging are visible (Figure 1A). Similarly, Western blot analysis revealed the presence of KIN-19, DAF-21/HSP90, and PAR-5 in the insoluble fraction from young *gon-2(−)* and *fem-1(−)* animals (Figure 3 and Figure S5C). In addition, our mass spectrometry analysis showed that the vast majority of both *fem-1(−)* and *gon-2(−)*/*glp-1(−)* age-aggregated proteins were also insoluble to some extent in the young animals (Figure 1B). Overall, the presence of insoluble proteins in the younger animals, albeit at relatively low levels, suggests that protein aggregation is not limited to mid-life or old animals but already occurring in healthy young animals. It is conceivable that these insoluble proteins have a function in young adults, but these findings are also consistent with recent reports showing that a decline in proteostasis begins in young *C. elegans* animals [40].

### Reduced Insulin/IGF-1 Signaling Can Delay and Prevent Inherent Protein Aggregation

**Figure 5:**
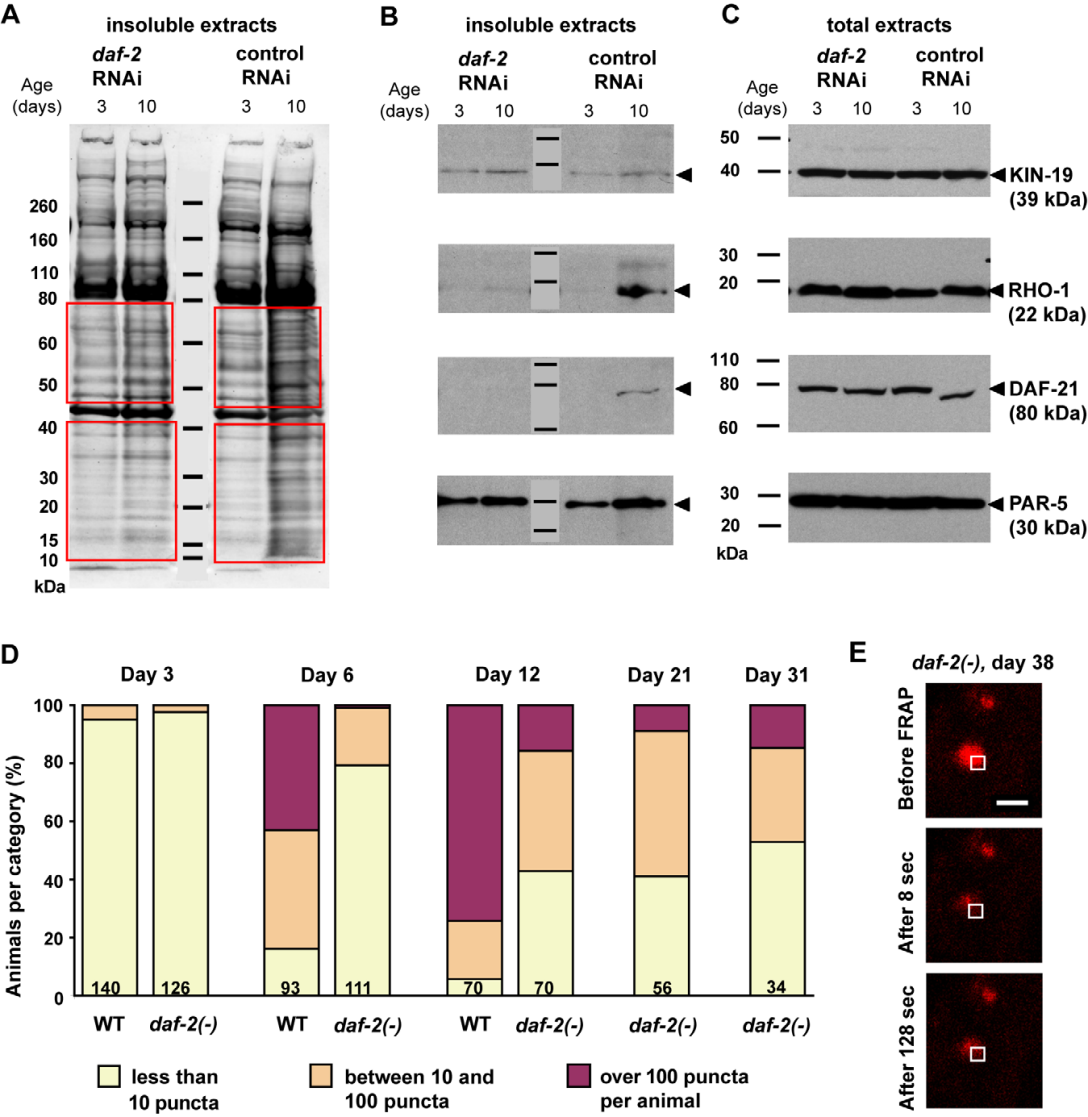
Reduced insulin/IGF-1-like signaling protects against age-dependent protein insolubility and aggregation. (A) Sypro Ruby staining revealed a decrease in overall age-dependent protein insolubility in *gon-2(−)*/gonad-less animals treated with *daf-2* RNAi compared to control RNAi (1.6-fold compared to a 3.6-fold increase with age, quantified in the red outlined areas). *daf-2* RNAi treatment prevented the insolubility of multiple proteins that appear with age in the total staining of insoluble proteins in control animals. (B) Western blot detection of specific candidates showed a slight delay in insolubility or the absence of insolubility in animals treated with *daf-2* RNAi. Quantification of the Western blots: KIN-19: *daf-2* RNAi, 2-fold increase with age; control RNAi, 3.4-fold. PAR-5: *daf-2* RNAi, 1.8-fold increase with age; control RNAi, 2.1-fold. (C) Decreased insolubility with reduced insulin/IGF-1 signaling is not correlated with a decrease in total protein levels of these proteins. Interestingly, *daf-2* RNAi treatment prevented the shift in size of DAF-21 in older animals. (D–E) The strong mutation *daf-2(e1370)* prevented KIN-19::tagRFP aggregation in the pharynx. (D) *daf-2(e1370)*; *Pkin-19::kin-19::tagrfp* animals had significantly fewer KIN-19::tagRFP puncta in their anterior pharyngeal bulbs than did wild-type animals expressing *Pkin-19::kin-19::tagrfp* (Day 6: *p*<0.0001, Day 12: *p*<0.0001, Yates' Chi-square test). No further increase in the number of puncta was observed after Day 12 in the *daf-2(e1370)* background, suggesting that reduced insulin/IGF-1-like signaling somehow caps the process of KIN-19::tagRFP puncta formation. The number of animals is indicated in the bars. (E) KIN-19::tagRFP puncta remained mostly soluble in a *daf*-2 mutant background. FRAP analysis of a KIN-19::tagRFP puncta in the anterior pharyngeal bulb of *daf-2(e1370)*; *Pkin-19::kin-19::tagrfp* animal, Day 38 (Laser setting: 10% in 0.8 µm^2^). As with this example, most KIN-19::tagRFP puncta present in a *daf*-2 mutant background uniformly lost fluorescence in the whole punta when bleached in a restricted area (Table 1). These results suggested that KIN-19::tagRFP does not aggregate in these puncta. Scale bar: 2 µm.

To further evaluate the importance of the aging process in mediating protein aggregation, we asked whether a mutation that slows aging would affect the rate of inherent protein aggregation. Mutation of the *daf-2*/insulin/IGF-1-receptor gene doubles the lifespan of *C. elegans* [41] and delays proteotoxicity in *C. elegans*' disease models of aberrant protein aggregation [42],[43]. Therefore we tested whether down-regulation of the insulin/IGF-1 pathway would delay inherent protein aggregation. As strong reduction-of-function mutations affecting insulin/IGF-1 signaling can influence reproduction [41],[44],[45], we chose to focus on aggregation in somatic tissues in the *gon-2(−)* mutants. When *daf-2* RNAi treatment is initiated at hatching or at the last larval stage, the animals grow to become long-lived adults [46]. The pattern of insoluble proteins in young-adult animals subjected to *daf-2* RNAi (from the first larval stage) resembled that of wild type (Figure 5A). However, we found only a slight increase in insolubility with age (1.6-fold) compared to control animals (3.6-fold). More strikingly, the insolubility pattern of proteins extracted from *daf-2(RNAi)* animals did not change with age, whereas the two patterns (young versus old) were very different in wild type. Exposure to *daf-2* RNAi from the last larval stage produced very similar results (unpublished data). Similarly, Western blot analysis showed that *daf-2* RNAi treatment greatly reduced the extent of both RHO-1 and DAF-21 insolubility with age and moderately reduced the extent of KIN-19 and PAR-5 age-dependent insolubility (Figure 5B).

The differences between protein insolubility in *daf-2(RNAi)* and control-RNAi animals could potentially have been caused by different levels of total protein available to aggregate. However, we found no change in KIN-19 or PAR-5 total levels and a slight increase in RHO-1 and DAF-21 total levels in aged *daf-2(RNAi)* animals compared to aged control animals (1.4-fold) (Figure 5C). Therefore, it is unlikely that reduced levels of protein insolubility produced by *daf-2* RNAi treatment was the result of less total protein. Overall, these results suggest that reduction in insulin/IGF-1 signaling specifically prevents the increase in protein insolubility that normally occurs with age without affecting the pattern of protein insolubility already present (in gel-based detectable amounts) in younger animals.

We also examined the effect of *daf-2* inhibition on protein aggregation in vivo, focusing on KIN-19. A relatively strong *daf-2* mutation, *e1370*, significantly delayed KIN-19::tagRFP puncta formation in the pharynx (Figures 2H and 5D). At Day 12, relatively few puncta were present in the long-lived mutants, whereas nearly all of the *daf-2(+)* animals had high levels of puncta. Unexpectedly, reduced insulin/IGF-1 signaling protected these animals against the formation of any further puncta after Day 12, even to Day 31, at which time all wild-type animals were dead and the *daf-2* mutants were beginning to die. Moreover, the puncta in the *daf-2(−)* mutant tended to contain mobile KIN-19 (even in very old Day 38 animals) (Figure 5E and Table 1). Indeed, FRAP analysis of 12- to 15-d-old animals revealed mobile KIN-19::tagRFP protein in 28 out of 34 puncta examined in the *daf-2(−)* mutant background compared to only 11 out of 36 puncta in the *daf-2(+)* animals (Fisher's exact test *p*<0.0001). These findings imply that the *daf-2(e1370)* insulin/IGF-1-receptor mutation protects against KIN-19 aggregation not only by slowing the rate of aging but also by preventing the aggregation process itself.

Although inhibition of DAF-2 signaling did not lead to a reduction in total aggregation-prone protein levels, by comparing our list of insoluble proteins to lists of genes identified in microarray analysis of long-lived *daf-2* mutants versus slightly short-lived *daf-16*; *daf-2* double mutants (*daf-16* encodes a FOXO-family transcription factor required for the longevity of *daf-2*-pathway mutants) [47], we found that *daf-2(e1370)* mutants had relatively low levels of transcripts encoding certain aggregation-prone proteins. In particular, we found that proteins prone to age-dependent insolubility were significantly over-represented among genes down-regulated in *daf-2(−)* mutants and under-represented among genes up-regulated in *daf-2(−)* mutants (Table S3, chi-square test *p* = 0.0002). Together, our findings suggest that reduced insulin/IGF-1 signaling could prevent protein aggregation in two ways: by promoting protein solubility and, for certain proteins, by modulating transcription.

### Protein Aggregation Occurs at Diverse Subcellular Localizations

**Figure 6:**
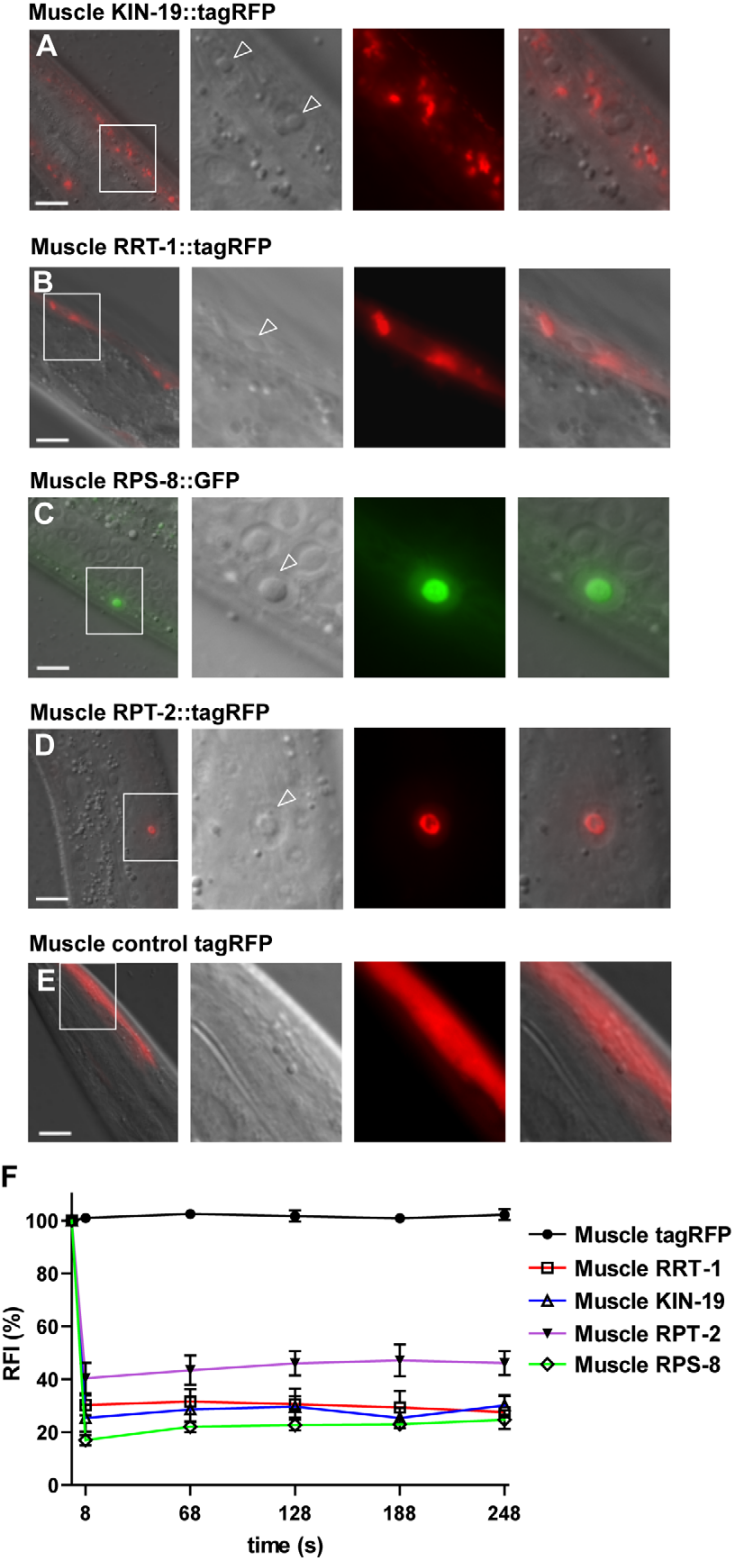
Aggregation occurs in many regions of the cell. (A–E) Localization of aggregation-prone candidates and control tagRFP expressed in body-wall muscle cells using the *myo-3* promoter. First panel shows Nomarski photograph overlayed with the fluorescent view for each aggregation-prone candidate. Scale bar: 10 µm. The next three panels show an enlargement of the boxed area in the first panel. Muscle nuclei are indicated in the enlarged Normarski photograph by open arrowheads. To obtain sufficient resolution with Normarski, we examined animals either at the last stage of development (L4) or as young adults. Comparison of Normarski and fluorescent photographs show that KIN-19 and RRT-1 formed puncta in the cytoplasm (A and B), whereas RPS-8 and RPT-2 aggregated in the nucleolus (C and D). Control tagRFP expressed alone in the muscle was diffusely localized throughout the muscle cells (E). (F) FRAP assay in puncta formed by the muscle-expressed aggregation-prone candidates demonstrated that these puncta contained immobile protein consistent with a state of aggregation. Muscle tagRFP remained mobile. Five puncta were evaluated in four to five young animals by FRAP for each aggregation-prone candidate.

To examine the subcellular localization of inherent protein aggregates, we expressed a selection of candidates in the same cell type, the body-wall muscle cells, using the *myo-3* myosin promoter. *C. elegans* body-wall muscle has been the tissue of choice for the expression of disease-aggregating proteins such as amyloid-β and polyglutamine repeats [42],[48] and is relatively easy to visualize without considerable autofluorescence build-up with age. We observed the presence of puncta during development and adulthood of the aggregation-prone candidates KIN-19, arginyl-tRNA synthetase (RRT-1), a ribosomal subunit (RPS-8), and a proteasomal protein (RPT-2) (Figure 6A–D). These puncta contained immobile protein, as analyzed by FRAP (Figure 6F). We note that both ribosomal proteins and aminoacyl-tRNA synthetases have been shown to have a relatively low mobility when present within the nucleolus [49],[50]. However, we observed no recovery 240 s after photobleaching, a time frame that should allow full recovery for a low-mobility protein. It was noteworthy that these proteins all formed insoluble puncta during development when overexpressed in muscle. This finding indicates that these proteins are capable of aggregating in very young animals, at least in this tissue. Similarly, disease-aggregating proteins such as expanded pathological polyglutamine repeats (over 35 polyglutamine repeat expansions) aggregate in the muscle during development [37],[42]. The lack of aggregation of the fluorescent tag alone when over-expressed in the muscle (Figure 6E) suggests that this early aggregation is related to the aggregation propensity of the candidates examined. We also expressed the aggregation-prone protein dynein light-chain 1 (DLC-1) fused to tagRFP in the muscle, but this fusion protein remained soluble. Because DLC-1 is a very small protein (only 10.3 kDa), it is possible that the much larger fluorescent tagRFP (27 kDa) masked the aggregation propensity of this protein.

As the integrity of muscle tissue degenerates sharply with age [51],[52], intracellular localization of age-dependent aggregates becomes challenging. Therefore, early aggregation in the muscle gave us the opportunity to employ differential-interference contrast microscopy to determine the positions of the puncta in the cell. We observed KIN-19 aggregation throughout the cytoplasm but not in the nucleus (Figure 6A). RRT-1/tRNA-synthetase was located diffusely throughout the muscle and accumulated in regions adjacent to the nucleus (Figure 6B) possibly in the proximity of the endoplasmic reticulum or in the aggresome. [We note that several known components of the aggresome, such as intermediate filaments (IFA-1, IFA-3, IFB-2), dynein (DLC-1, DLC-2, DHC-1), and 14-3-3 (PAR-5) [53] were identified among the proteins prone to aggregate with age.] RPS-8 was present at low levels throughout the cytoplasm and nucleoplasm and accumulated to form a bright mass in the nucleolus (Figure 6C). Ribosomal subunits localize to the nucleolus to assemble with rRNA and other subunits to form the mature ribosome. Therefore, aggregation at the nucleolus suggests that ribosomal subunits could be aggregating as FRAP-insoluble pre-ribosomal particles. Finally, we found that the proteasomal 19S protein RPT-2 formed FRAP-insoluble ring-like structures in the nucleolus (Figure 6D). Under normal conditions, proteasomes are found in the nucleoplasm and are not localized to the nucleolus [54]. Therefore, their aggregation could lead to their mislocalization to the nucleolus. It is possible that the cellular sites of aggregation differ between young and old animals. However, we observed a very similar pattern of intracellular KIN-19 puncta localization during development (in muscles) and in old animals (in the pharynx), suggesting that aggregation may occur in the same cellular locations in young and old animals.

Overall, our results suggest that inherent protein aggregation is not restrained to a single subcellular localization such as the aggresome but that multiple centers of aggregation exist, potentially affecting different cellular processes.

### Muscle KIN-19 Aggravates Paralysis Caused by Polyglutamine Repeats

**Figure 7:**
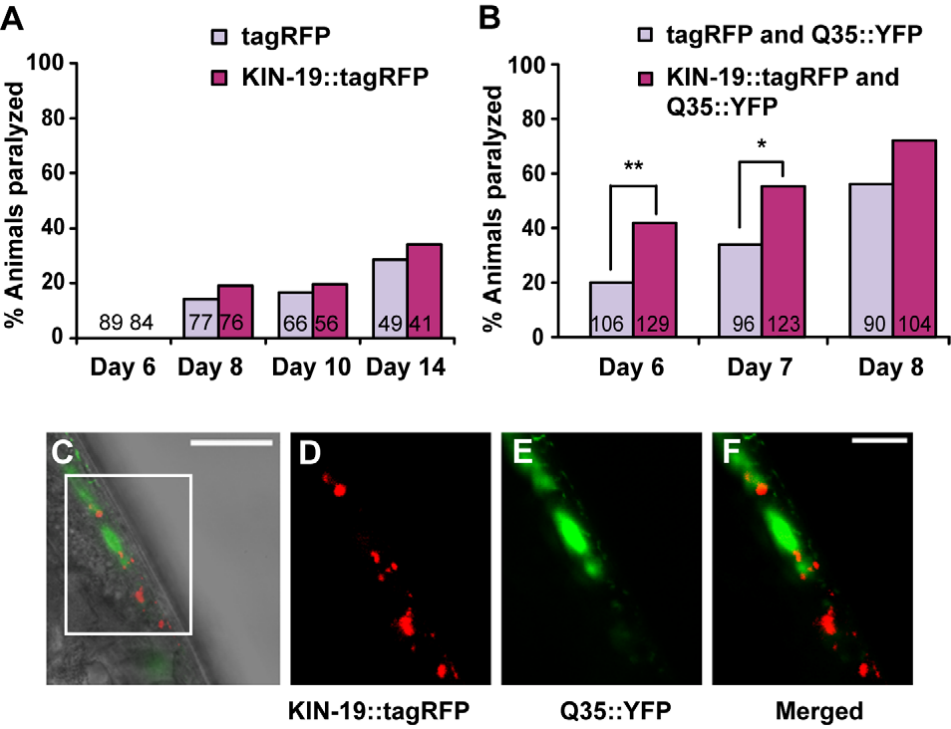
Muscle KIN-19::tagRFP accelerates the paralysis caused by polyglutamine-repeat proteins. (A) Animals expressing *Pmyo-3::kin-19::tagRFP*, which exhibit KIN-19::tagRFP aggregates in the muscle, were not more likely to become paralyzed with age than were animals expressing only tagRFP. (B) On the first day of paralysis with Q35 (Day 6), 42% of animals also expressing KIN-19::tagRFP in the muscle were paralyzed, compared to only 19% of control animals expressing the tagRFP reporter (Yates' Chi-square test, ** *p*<0.0005). A significant difference was also observed at Day 7 (Yates' Chi-square test, * *p*<0.01) but not at Day 8. (A–B) The number of animals is indicated in the bars. For the five additional trials we performed, see Table S4. (C–F) Muscle-aggregated KIN-19 and Q35 do not co-aggregate. Overlay of bright-field and fluorescent images of adult muscle (Day 3), scale: 50 µm (C). Enlarged boxed area with KIN-19::tagRFP in red (D), Q35-YFP in green (E), and overlay (F), scale: 10 µm.

Expressing disease-associated aggregation-prone proteins such as polyglutamine-repeat proteins in the muscle paralyzes the animal as it ages [42],[55]. As described above, fluorescent-tagged KIN-19 aggregated abundantly throughout the muscle cells in a similar fashion to polyglutamine repeats or amyloid-β [42],[56] (Figure 6A). Therefore we asked whether these animals would become paralyzed with age. However, we found that the increased levels of KIN-19 aggregation in these animals did not cause paralysis (Figure 7A). Therefore it was particularly interesting to test whether KIN-19 aggregation in muscle cells also containing polyglutamine-repeat proteins could enhance polyglutamine-repeat-related pathology. To examine this possibility, we expressed *Pmyo-3::kin-19::tagrfp* in animals expressing 35 polyglutamine repeats (Q35) under the control of the muscle-specific *unc-54* promoter [42]. Q35 transgenic animals exhibited an age-dependent paralysis beginning on Day 6 of adulthood. As KIN-19 formed aggregates in the muscle before the onset of Q35 aggregation, we asked whether KIN-19 aggregation could act as a seed for Q35 aggregation. However, we found that Q35 and KIN-19 puncta were distinct, so the two proteins clearly did not co-aggregate (Figure 7C–F). Another possibility was that high levels of aggregation-prone KIN-19 would inhibit the cellular proteostasis systems, thereby increasing the number of Q35 aggregates, as appears to occur when misfolded temperature-sensitive mutant proteins are co-expressed with Q35 [19]. We evaluated the numbers of large visible Q35 aggregates in 3-d-old adult Q35 transgenic animals containing KIN-19 aggregates compared to Q35 control animals over-expressing tagRFP alone. Surprisingly, we found a slight, albeit statistically significant, decrease in Q35 aggregates in animals with the aggregation-prone protein (Figure S6, Kruskal-Wallis test, *p*<0.0001). Despite no large changes in levels of polyglutamine-repeat aggregation, increased levels of KIN-19 aggregation in the muscle caused at least twice as many Q35 transgenic animals to become paralyzed by Day 6 in several trials (Figure 7B, Table S4). No significant difference in paralysis was observed at the advanced disease-state 2 days later. Taken together, the data suggest that KIN-19 specifically modulates the initial paralysis threshold in Q35-containing animals. As a consequence, increasing the level of this aggregation-prone protein impacts the pathology caused by disease aggregating polyglutamine-repeats.

### Specific Functional Protein Categories Are Prone to Aggregate with Age

In addition to examining the properties of several insoluble-prone proteins in vivo, we looked within the entire set of aggregation-prone proteins for common functional or structural features. Using the NIH-DAVID software [57], we found that many of the proteins present in our insoluble fraction were proteins that are known to function early in life, where they play an important role in embryonic development, growth, translation, and protein homeostasis (Table 2). These categories could potentially represent proteins aggregating more specifically in the oocytes. However, we found an over-representation of developmental processes among the top 25th percentile of proteins prone to aggregate with age in the somatic tissues of strains lacking oocytes (*p* = 2.3E-5; unpublished data). Furthermore, DAVID analysis of SwissProt and PIR terms revealed a significant over-representation of the proteasome (21 proteins, EASE *p* = 3.8E-10), ribosomal proteins (62 proteins, EASE *p* = 6.6E-28), and chaperones (17 proteins, EASE *p* = 3.2E-5), including HSP90/DAF-21, an HSP70 (HSP-1), several small heat shock proteins (HSP-16.11, HSP-16.49, and SIP-1), as well as six chaperonin subunits. These cellular components ensure proteostasis in young animals and thus explain in part the over-representation of growth and translation GO categories among aggregation-prone proteins.

**Table 2:** Specific functional categories are over-represented in the whole aggregation-prone protein set.

Gene Ontology Term	Number of Proteins	% of Total	*p* Value
Embryonic development	334	51%	1.1E-43
Translation	100	15%	6.3E-42
Growth	257	39%	1.3E-23
Cofactor metabolic process	38	6%	1.2E-08
Protein folding	31	5%	2.2E-07
Cellular respiration	12	2%	3.2E-07
tRNA aminoacylation	18	3%	5.3E-07
Determination of adult life span	30	5%	1.2E-04
Cytokinesis	24	4%	1.3E-04
Cytoskeleton organization and biogenesis	32	5%	4.6E-04

Functional annotation of the aggregation-prone protein set was carried out using the DAVID software. A total of 657 out of 711 aggregated proteins were recognized by DAVID and 492 of these fell into one or more significant gene ontology biological process category. EASE score *p* value: modified Fisher Exact *p* value.

Comparing functional categories over-represented in the age-dependent and age-independent insoluble sets, we found overall similar categories (Table S5A–B). However, proteins responsible for protein folding and presumably normally soluble proteins such as RHO-1 that regulate cytoskeleton organization were specifically enriched in the age-dependent insoluble set, whereas proteins related to cellular respiration were only enriched in the age-independent insoluble set.

Interestingly, we found an over-representation of the GO “determination of adult life span” term, suggesting that aggregation-prone proteins tend to play a role in the aging process. If the presence of aggregation-prone proteins is detrimental to the organism, we should also observe an over-representation of these candidates in RNAi screens previously carried out to identify factors whose inhibition increases lifespan. We observed a significant overlap between genes found to prolong lifespan when inhibited after development and genes encoding proteins prone to aggregate with age (Table S6, 11 out of 56 genes, cumulative hypergeometric probability: *p*(X≥11) = 0.0003) [58]. We found an even larger overlap when comparing to the whole insoluble set (Table S6, 18 out of 56 genes, cumulative hypergeometric probability: *p*(X≥18) = 5.8E-7). In addition, we observed a significant overlap between the whole insoluble protein set and a genome-wide RNAi library screen for longevity genes (Table S6, 9 out of 27 genes, cumulative hypergeometric probability: *p*(X≥9) = 0.0003) [59]. The products of these genes included proteins that function in translation and mitochondrial respiration, whose inhibition is known to increase lifespan [60],[61],[62]. In addition, the set included nine genes not directly related to these two main categories. For example, we found *gex-15*, a gene encoding a GEX-3 interacting protein [63], which plays a role in development [64]; *maoc-1*, which encodes Mao-c-like dehydratase domain protein 1, predicted to function in peroxisomal fatty acid beta-oxidation; *sams-1*, which encodes S-adenosyl methionine synthetase, a protein that functions as a universal methyl group donor; and *pat-6*, a gene encoding actopaxin, which binds an integrin-linked kinase [65].

Overall, functional similarities among aggregation-prone proteins, in particular proteins related to proteostasis regulation, show that the process of aggregation is not random. The significant overlap between aggregation-prone proteins and proteins whose inhibition increases lifespan raises the possibility that the process of aggregation itself tends to negatively impact the organism.

### Aggregation-Prone Proteins Are Distinct in Structure from the Proteome

**Figure 8:**
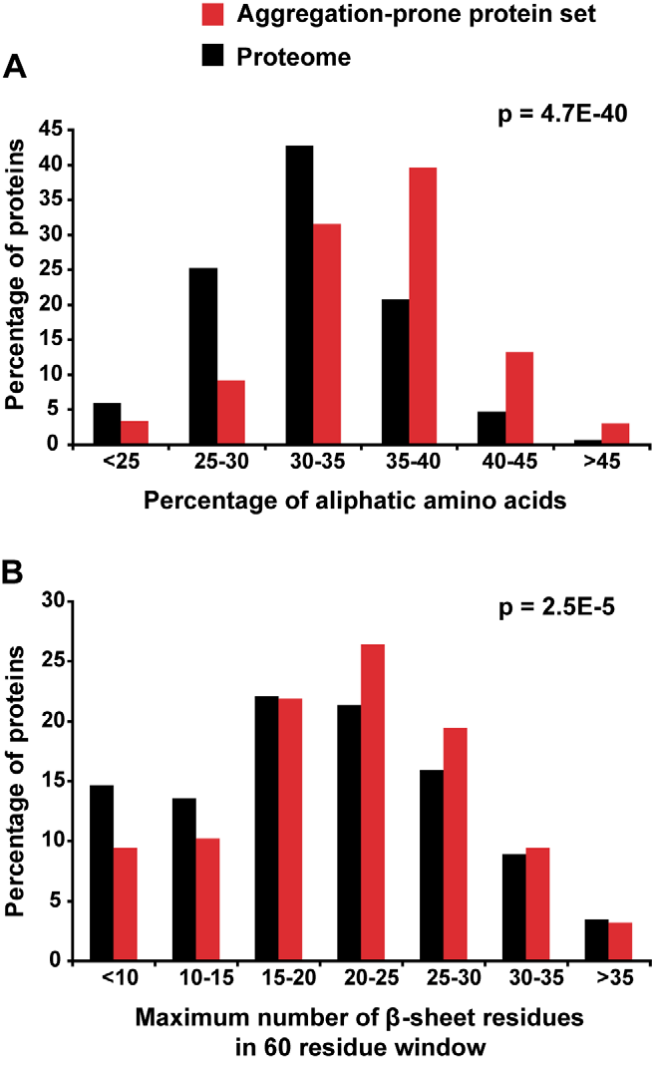
Aggregation-prone proteins are enriched in aliphatic amino acids and extended stretches of β-sheet propensity. (A–B) Bioinformatic analysis of aggregation-prone proteins (red) compared to the total set of *C. elegans* proteins detected by mass spectrometry (black). (A) Aggregation-prone proteins were significantly enriched in aliphatic residues (*p* = 4.7E-40) as evaluated by an unequal variance *t* test. (B) Scanning window analysis showed that aggregation-prone proteins are enriched in long stretches of β-sheet propensity (unequal variance *t* test *p* = 2.5E-5).

To determine whether proteins prone to aggregate exhibit sequence or structural properties that distinguish them from the rest of the *C. elegans* proteome, we compared amino acid residue composition, predicted secondary-structure content, and fold classification. As a background proteome dataset, we compiled a list of all *C. elegans* proteins detected in mass spectrometry experiments available in PeptideAtlas [66]. This allowed us to avoid potential bias caused by including proteins that would not be normally detected by mass spectrometry. However, we note that all our results are also significant if using the whole *C. elegans* proteome (unpublished data). First, examining amino acid composition, we observed a significant enrichment in aliphatic amino acids among aggregation-prone proteins—in particular, alanine, glycine, and valine (Figure 8A). Conversely, several amino acids were significantly under-represented—in particular, proline, which disrupts secondary structures such as β-sheets (Figure S7A). Next, we predicted the secondary structure for each protein using PSIPRED [67]. We observed a highly significant increase in β-sheet content (Figure S7B) but no difference in α-helical content (Figure S7C) in aggregation-prone proteins relative to the proteome. Additional scanning window analysis showed that aggregation-prone proteins are significantly enriched in long stretches of β-sheet propensity—in particular, 20–30 amino acid stretches (Figure 8B).

To identify any biases in the aggregation-prone proteins toward particular tertiary structures, we assigned fold classifications to each *C. elegans* protein with significant sequence homology to a known structure in the CATH database, which is a comprehensive hierarchical classification of protein domain structures [68]. This revealed a significant structural bias in the aggregation-prone proteins relative to the proteome towards folds with β-sheets (Figure S7D). In particular, we found a small enrichment in proteins containing mixed α-helix and β-sheet folds as well as β-barrel folds. Conversely, proteins with orthogonal bundle folds (CATH 1.10), which are α-helix rich, were under-represented among aggregation-prone proteins.

Finally, we evaluated whether age-dependent insolubility was linked to a different profile in amino acid composition or predicted secondary structure compared to age-independent insolubility. We found that structural differences identified in the whole insoluble set were conserved in both the age-dependent and age-independent insoluble set (Table S7). In general, these structural enrichments tended to be slightly less significant in the age-dependent compared to the age-independent insoluble set. In particular, age-dependent insoluble proteins had significantly less glycine compared to age-independent insoluble proteins.

Together, our analysis indicated that protein insolubility and age-dependent aggregation propensity is correlated with an enrichment of specific residues and increased β-sheet propensity.

## Discussion

### Widespread Protein Insolubility Occurs in a Multicellular Organism

A small number of aberrant aggregating proteins have been associated with disease. Our results demonstrate, for the first time to our knowledge, that several hundred proteins become insoluble during normal aging in the multicellular organism, *C. elegans*. A few of these proteins, such as gamma-tubulin [30], are known to be functional in the cell in an insoluble state. However, the majority of the proteins that we identified are functional in a soluble state. Therefore, we believe that they are becoming misfolded with age and aggregating. Indeed, studies have shown that many proteins have the potential, when misfolded, to aggregate via self-complementary sequences [9],[10]. Consistent with this, we observed the aggregation of five out of six fluorescently tagged proteins that we examined in vivo (the exception being a protein that was much smaller than the fluorophore). Conversely, the majority of age-insoluble proteins were maintained as soluble by reducing *daf-2*/insulin/IGF-1 signaling, which also extends lifespan, again arguing against these proteins' being functional in the insoluble state.

### Inherent Protein Aggregation Increases with Age

Temperature-sensitive mutant proteins are prone to misfold even in healthy young animals; thus a decline in proteostasis begins at a relatively early age in *C. elegans* [40]. Likewise, we observed large numbers of insoluble protein species in relatively young animals (Day 3 of adulthood at 25°C). Quantitative mass spectrometry and Western blot analysis revealed a considerable increase in protein insolubility in older animals. We found that an increase in the overall level of a protein is not necessary to induce age-dependent protein insolubility and aggregation. Future experiments will be required to learn whether age-dependent protein aggregation is caused by increased levels of damage in long-lived proteins over time and/or by a change in the cellular environment.

Our analysis of four candidates suggests that approximately 10% of the aggregation-prone protein available becomes highly insoluble in aged somatic tissues. Our extraction method does not distinguish soluble and membrane-bound proteins from soluble oligomers, which could potentially nucleate the formation of large, insoluble aggregates. Further investigation into the oligomer levels of these proteins will be needed to give a better estimate of the proportion of non-functional aggregation-prone proteins in the cell.

### Inherent Protein Aggregation Is Modulated by the Insulin/IGF-1 Signaling Pathway

Reducing insulin/IGF-1 signaling is known to trigger the expression of many cell-protective proteins and processes [69],[70],[71],[72] that prevent the collapse of proteostasis. Reduced insulin/IGF-1 signaling also improves the paralysis phenotype caused by disease-aggregating proteins such as polyglutamine repeats and amyloid-β [42],[43]. Suppression of insulin/IGF-1 signaling by RNAi was sufficient to prevent or greatly reduce age-dependent protein insolubility in aged animals at a time point when half the control animals are dead. Life-long and stronger suppression of insulin/IGF-1 signaling changed the nature of the aggregation process, as we found the majority of KIN-19::tagRFP-labeled puncta to contain mobile protein even near the end of these long-lived animals' lives. Interestingly, these results suggest that at least for KIN-19, the puncta could be delimited by a structure such as membrane or intermediate filament network to prevent diffusion of mobile KIN-19 away from the puncta. One intriguing possibility is that conditions that inhibit insulin/IGF-1 signaling stimulate the recruitment of cellular factors to these puncta that maintain physically associated aggregation-prone proteins in a soluble state.

Another possibility to explain the decrease in protein insolubility mediated by reduced insulin/IGF-1 signaling is that the transcription of the corresponding genes is down-regulated, thereby reducing the total levels of protein available to aggregate. When grown at 25°C, *daf-2(e1370)* mutants enter the dauer state during development, which is associated with reduced protein synthesis. However, we showed previously that the global rates of protein synthesis are not reduced in *daf-2*(*e1370*) mutants grown at 20°C [60], where the animals do not become dauers. *daf-2(RNAi)* animals grown at 25°C do not become dauers, thus resembling *daf-2(e1370)* animals grown at 20°C. We did not observe a change in total protein levels detected by Sypro ruby in *daf-2(RNAi)* animals grown at 25°C (unpublished data). Moreover, none of the proteins we examined individually by Western blot was present at lower levels in *daf-2(RNAi)* animals than in control animals. Thus mechanisms that do not involve reductions in the levels of specific aggregation-prone proteins levels must influence their aggregation. Nevertheless, it was intriguing to find that *daf-2(−)* mutants preferentially down-regulate the transcription of certain genes encoding proteins prone to insolubility with age. This finding raises the novel possibility that part of the longevity of *daf-2* mutants is due to the reduced level of aggregation of certain proteins, rather than simply to the reduced levels of their normal cellular functions. Mechanistically, it would be particularly interesting to investigate whether autophagy, which increases in *daf-2* mutants, protects against specific protein aggregation, as is the case for β-amyloid aggregation in these mutants [73]. It will also be interesting to learn whether mutants whose lifespans are extended for other reasons, such as reduced TOR or respiration levels, also have reduced levels of aggregated proteins.

### Aggregation-Prone Proteins Regulate Proteostasis and Prevent Disease Protein Aggregation

Interestingly, we found that age-dependent protein insolubility affects a wide variety of systems involved in maintaining proteostasis. We identified major chaperones such as HSP90/DAF-21 and an HSP70 (HSP-1), several small heat shock proteins, and several chaperonin subunits. We also found that a significant fraction of the proteasome subunits are prone to aggregate with age. Chaperones and the proteasome play a major role in preventing the accumulation of misfolded proteins and are directly implicated in preventing protein aggregation [13],[74],[75],[76].

We also identified a significant fraction of ribosomal proteins among our aggregation-prone proteins. Subcellular localization showed that ribosomal proteins aggregate in the nucleolus. This aggregation could potentially reduce the number of mature ribosomes available to perform translation. Indeed, ribosomal protein aggregation caused by reductive stress is associated with decreased protein synthesis [77]. In addition, we found an over-representation of mitochondrial proteins, among which were several ATP synthase subunits, although these tended not to become more insoluble with age. The significance of this insolubility is not completely clear. Mitochondrial dysfunction has been implicated in several neurodegenerative diseases [78],[79],[80] and maintaining ATP levels has been suggested to protect against α-synuclein aggregation [81]. Conversely, inhibiting respiration and ATP synthase activity can have the beneficial effect of extending lifespan.

It remains unclear why so many essential proteins involved in proteostasis become aggregation-prone during the aging process. One possibility is that these proteins aggregate as a consequence of their interaction with misfolded aggregation-prone protein substrates. However, it has been demonstrated previously that chaperones can interact transiently with polyglutamine aggregates without aggregating themselves [82]. Furthermore, the proteasome remains active even if associated with aggregated proteins at the aggresome [83].

As multiple aggregation-prone proteins play an important role in maintaining proteostasis, their aggregation could potentiate the aggregation of other proteins, including disease-aggregating proteins. Indeed, subunits of the proteasome, ribosome, and chaperonin were identified among the RNAi clones in a genome-wide screen that were reported to increase polyglutamine aggregation in *C. elegans* [20]. In fact, we found 69 of the 156 proteins (44.9%) identified in this RNAi screen and represented in our compiled mass spectrometry proteome were among our set of aggregation-prone proteins (Table S8, cumulative hypergeometric probability *p*(X≥69)≈0). Forty-six of these proteins were age-dependent aggregation-prone proteins. The overlap between aggregation-prone proteins and suppressors of polyglutamine aggregation supports the notion that inherent age-dependent protein aggregation would impact protein-aggregation disease.

### Inherent Protein Aggregation Aggravates the Pathology Caused by Polyglutamine-Repeat Aggregation

In this study, we analyzed the age-dependent aggregation of KIN-19, the *C. elegans* homolog of casein kinase (CK)1α, which has been identified in tau deposits in both Alzheimer's disease and sIBM [33],[34]. Our results shed new light on this observation by implying that CK1α could itself be aggregating with age in the nervous system and the muscle, as occurs in *C. elegans*. We found that KIN-19 was capable of influencing disease-aggregation protein toxicity even in the absence of any detectable co-aggregation. Specifically, we found that animals co-expressing KIN-19 and Q35 did not have increased Q35 aggregate levels and yet displayed increased paralysis. Why might this be? Many recent studies show that low-MW oligomers formed by disease-misfolded proteins are at least as, if not more, toxic as high-MW, visible, aggregates [43],[84],[85],[86]. The presence of aggregation-prone KIN-19 in the muscle could enhance the formation of toxic low MW intermediate species of Q35 in at least two ways: First, misfolded KIN-19 could sequester chaperones and/or impair the degradation system. Second, misfolded aggregation-prone KIN-19 could overload a system that favors aggregate formation, such as the aggresome, or it could sequester a pro-aggregation factor. Both possibilities would result in more toxic low-MW oligomers, causing enhanced paralysis. Finally, KIN-19 could influence Q35 paralysis through its enzymatic function as a protein kinase. However, over-expression of KIN-19 in the muscle did not cause increased paralysis, indicating that it is not toxic in otherwise normal animals. Moreover, in mammalian cells, CK1α/KIN-19 promotes cellular survival and enhances the response to certain stressors [87],[88],[89]. Therefore, factors associated with KIN-19 aggregation may be more relevant in this situation, especially as the property of aggregation is prominent and shared between KIN-19 and Q35.

### Aggregation-Prone Proteins Tend to Modulate Lifespan

In principle, increased protein aggregation with age could have a beneficial effect by sequestering damaged proteins and toxic low-MW oligomers. Alternatively, they could contribute to the aging process by reducing the cell's capacity for proteostasis, triggering a vicious cycle that leads to further protein misfolding and aggregation. Intriguingly, we found a highly significant overlap between our aggregation-prone proteins and proteins whose reduction increases lifespan. These included (but were not restricted to) proteins involved in translation and mitochondrial respiration [58],[59]. Our findings offer a new possible explanation for the longevity of these animals, namely, that the aggregation process is itself toxic and therefore, preventing the aggregation of these proteins by reducing their levels could protect the organism.

### Structural Characteristics of Aggregation-Prone Proteins

Disease-related protein aggregation is characterized by amyloid fibrils consisting of a cross-β structure formed by the association of identical proteins [86]. Hydrophobic residues and β-sheets are known to be critical determinants of amyloid formation [86], and both were over-represented in the insoluble proteins identified in *C. elegans*. The over-representation of the hydrophobic residue valine could in part explain the increased levels of predicted β-sheets in these proteins. In addition, we found a highly significant under-representation of prolines in aggregation-prone proteins. Proline functions as an important structural modulator by disrupting secondary structures such as β-sheet strands. Therefore the longer stretches of β-sheets observed in aggregation-prone proteins could in part be the result of fewer prolines in the primary sequence. Although we cannot conclude whether inherently aggregated proteins are in an amorphous or structured aggregated form, our results are compatible with the idea that some aggregation-prone proteins may transition into an amyloid state. Indeed, these extended β-sheets could promote protofibrils that then could nucleate to form amyloid aggregates. Furthermore, for one of the candidates we examined on Western blots, DAF-21, we detected the presence of urea-insoluble material, indicative of a highly structured aggregate (Figure S5C).

A recent study showed that self-complementary sequences with high aggregation propensity are enriched in valine, alanine, isoleucine, and serine [10]. In addition, short sequences of six to eight residues have been shown to promote fibril formation in disease-aggregating proteins [90], some of which are enriched in glycine, alanine, and valine residues [91],[92]. Therefore, the overrepresentation of glycine, alanine, and valine residues in our insoluble protein set could promote their aggregation.

We note that the structural features that distinguish aggregation-prone proteins from other proteins were overrepresented in both age-dependent and age-independent insoluble proteins identified in somatic tissues. Because nearly all insoluble proteins became more insoluble with age in animals with oocytes, it is possible that all these proteins are prone to misfolding and aggregation with age. Therefore it would not be unexpected to find similar structural features. However it is also possible that age-dependent aggregation propensity can be explained by a structural or other characteristic yet to be discovered.

### Aggregation-Prone Proteins Identified in *C. elegans* Are Overrepresented in Human Disease Aggregates

An important question is whether age-related protein aggregation is an evolutionarily conserved process that also occurs in higher organisms and, if so, to what extent inherent protein aggregation can influence diseases related to aggregation. In the context of disease, aggregates are mainly composed of hallmark proteins such as β-amyloid, tau protein, huntingtin, and α-synuclein. However, mass spectrometry analysis of amyloid structures from neurodegenerated human brain tissue has shown that other proteins are present at lower amounts in these aggregates [16],[17],[18]. Strikingly, we found that homologs of 13 out of 24 proteins identified in amyloid plaques (*p*(X≥13) = 2.5E-8) and homologs of 32 out of 65 proteins identified in neurofibrillary tangles from Alzheimer's disease patients (*p*(X≥32)≈0) were prone to aggregate in *C. elegans* (Tables S9 and S10). Similarly, we observed that homologs of 11 out of 35 proteins discovered in Lewy bodies from patients with a Lewy body variant of Alzheimer's disease were aggregation-prone in *C. elegans* (*p*(X≥11) = 0.0002) (Table S11). In addition, detergent-insoluble proteins enriched in a mouse model for amyotrophic lateral sclerosis (ALS) showed striking overlap with *C. elegans* proteins with a propensity for insolubility [93]. The homologs of 26 out of 31 detergent-insoluble proteins accumulating in spinal cords from these mice were identified in the aggregation-prone protein set in *C. elegans* (*p*(X≥22)≈0) (Table S12). This remarkable overlap between insolubility in *C. elegans* and disease-dependent insolubility in mammals strongly suggests that the basic proprieties and underlying mechanisms causing these proteins to misfold and aggregate in *C. elegans* are evolutionary conserved, at least in the context of disease. These results raise the possibility that inherent protein aggregation might directly influence the aggregation and pathology of the main disease-aggregating proteins in humans.

### Conclusion

In summary, we have found that several hundred proteins, many of which are associated with cell growth, become aggregation-prone in *C. elegans* as it ages. Our findings suggest that the aging process itself can be a causative factor for protein aggregation and that reducing the rate of aging can prevent aggregation. Our results open a new field of research into the role of inherent protein aggregation in normal aging and in disease-related aggregation. Further investigation into the factors influencing the cell's normal protein-aggregation landscape will be important to understand the process of inherent protein aggregation and could lead to the identification of therapeutic targets for disease intervention. In addition, levels of inherent protein aggregation could be used as a new biomarker to evaluate the changes in proteostasis with age or in disease conditions.

## Material and Methods

Note: Age is defined by the number of days of adulthood starting from the last larval stage L4. Unless the number of days is mentioned, “aged” animals are defined as the time point when half of the population is alive.

### Strains

Wild type: N2.

### Mutants

CF2137: *fem-1(hc17) IV*, CF2253: *gon-2(q388) I*, CF1903: *glp-1(e2141) III*, CF1041: *daf-2(e1370) III*.

### Transgenics

CF3166: *muEx473[Pkin-19::kin-19::tagrfp+Ptph-1::gfp]*, CF3227: *daf-2(e1370) III*; *muEx473[Pkin-19::kin-19::tagrfp+Ptph-1::gfp]*, CF3317: *muEx512[Pkin-19::tagrfp+Ptph-1::gfp]*, CF3327: *muEx513[Pmyo-3::kin-19::tagrfp+Ptph-1::gfp]*, CF3328: *muEx514[Pmyo-3::rpt-2::tagrfp+Ptph-1::gfp]*, CF3549: *muEx515[Pmyo-3::rps-8::gfp+Podr-1::cfp]*, CF3505: *muEx563[Pmyo-3::rrt-1::tagrfp+Ptph-1::gfp]*, CF3330: *muEx516[Pmyo-3::tagrfp+Ptph-1::gfp]*, AM140: *rmIs132[Punc-54::q35::yfp]*, SA115: *unc-119(ed3) III*; *tjls1[Ppie-1::gfp::rho-1+unc-119(+)]*, CF3372: *rmIs132*; *muEx513*, CF3373: *rmIs132*; *muEx516*.

### Cloning and Strain Generation

Cloning was carried out using the Gateway system (Invitrogen, Carlsbad, CA, USA). Promoters and cDNA of the genes we used were obtained from Open Biosystems (Thermo Scientific, Huntsville, AL, USA). All constructs contain the *unc-54* 3′ UTR. The tagrfp vector was obtained from Evrogen (AXXORA, San Diego, CA, USA). Constructs were sequenced at each step. *Pkin-19::kin-19::tagrfp* or *Pkin-19::tagrfp* were injected at 5 ng/µl together with the coinjection marker, *Ptph-1::gfp* (at 50 ng/µl) into N2 animals. *Pmyo-3::kin-19::tagrfp*, *Pmyo-3::tagrfp*, *Pmyo::rpt-2::tagrfp*, *Pmyo::rps-8::tagrfp* were injected at 30 ng/µl together with the coinjection marker, *Ptph-1::gfp* (at 50 ng/µl) into N2 animals.

### Aggregation-Prone Protein Extraction

To obtain large synchronized populations of aged animals, we used temperature-induced sterile mutants. We chose not to use FUDR-induced sterility, as this treatment was not as effective in our hands as temperature-induced sterile mutants and resulted in morphological abnormalities in a fraction of animals. We note that the sterile strains were, to different extents, longer-lived than wild-type animals (Figure S8). It is possible that conditions that extend lifespan reduce aggregation, as we saw greatly reduced levels in the very long-lived *gon-2* mutants treated with *daf-2* RNAi (see text). Therefore, it is possible that the extent of protein aggregation may have been even greater in wild-type animals. Eggs were collected from adult animals and L1 arrest was performed overnight at 20°C. For *gon-2(ts)* animals, we transferred the parent animals at the L4 stage to 25°C. 3,200 L1s were distributed per 14 cm diameter plate with normal growth agar and kept at 25°C until collection to avoid temperature-dependent artifacts. ∼45,000 animals were collected at Day 3 of adulthood (young animals) or when half the animals were estimated to be dead (aged animals; evaluated by counting the alive/dead animal ratio in several areas of the plates to be collected).

#### Sucrose separation

Dead animals and bacteria were removed by flotation on a 30% sucrose solution. Animals were incubated in a nutator for another 30 min to eliminate all residual bacteria. Animals were washed rapidly once with RAB buffer (0.1 M MES, 1 mM EGTA, 0.1 mM EDTA, 0.5 mM MgSO_4_, 0.75 M NaCl, 0.02 M NaF) without protease inhibitors and then an equal volume of RAB buffer with Roche Complete Inhibitors 2× (Roche Molecular Biochemicals, Indianapolis, IN, USA) was added. Animals were immediately drip frozen in liquid nitrogen and ground to powder in a mortar.

#### Sequential extraction for proteomic analysis

The entire extraction procedure was carried out on ice and centrifugation steps were at 4°C. Because of losses during grinding, we choose to normalize by sample weight. If the young and old worms are the same size, then this does not cause any distortion. However, the older worms are very slightly smaller (see below) and assuming that they have not lost insoluble protein during the shrinkage (rather, for example, water or fat), then our estimation of the increase in insolubility with age is slightly higher than it should be. Indeed, measurements of length and width (at vulva) of Day 3 young adults compared to aged adults show that the width of the animal does not change with age, but the length decreases very slightly. *gon-2(−)*: aged adult length = 94.5%±6.9% of young adult length (Student's *t* test *p* = 0.04), width = 101.5%±12.9% of young adult width (Student's *t* test *p* = *ns*), *n* = 15; *glp-1(−)*: aged adult length = 91.2%±6.8% (Student's *t* test *p* = 0.002), width = 103.8%±9.9% (Student's *t* test *p* = *ns*), *n* = 15; *fem-1(−)*: aged adult length = 87.5%±5% (mean ± SD) (Student's *t* test *p*<0.001), width = 95%±13% (Student's *t* test *p* = *ns*), *n* = 15. 300 mg of young or aged animals were solubilized in two volumes of RAB (0.1 M MES, 1 mM EGTA, 0.1 mM EDTA, 0.5 mM MgSO_4_, 0.75 M NaCl, 0.02 M NaF) with Roche Complete Inhibitors 2×, DNaseI and RNaseI, and extracted using a syringe (27G1/2, Becton-Dickinson, Fischer scientific, Cincinnati, OH, USA). Cytosolic soluble proteins were removed by centrifugation at 20,000 g. The pellet was reextracted in RAB with 1 M sucrose to help remove lipids. The resulting pellet was extracted in RIPA buffer (50 mM Tris pH 8, 150 mM NaCl, 5 mM EDTA, 0.5% SDS, 0.5% SDO, 1% NP-40, 1 mM PMSF, Roche Complete Inhibitors 1×) three times using a syringe and centrifuged each time at 20,000 g to remove membrane proteins. This final pellet was solubilized in 70% formic acid and centrifuged at 50,000 g to remove worm cuticlar debris. The supernatant was collected and dialyzed on membrane filters disks, 0.025 µm pore size (Millipore, Billerica, MA, USA) against 50 mM Tris, 1 mM DTT, 0.1 mM PMSF, pH 7.5.

#### Extraction for whole protein stain and Western blot analysis of protein insolubility

For Western blot analysis, *fem-1(−)* animals grown on OP50 or *gon-2(−)* animals grown on control RNAi (targeting GFP or the vector L4440) or *daf-2* RNAi were collected in RAB buffer. To extract aggregated proteins, 50 mg of the frozen animals after sucrose separation were directly resuspended in 150 µl RIPA buffer (supplemented with Roche Complete Inhibitors 2×). RAB and RIPA soluble proteins were collected after centrifugation at 20,000 g, 4°C. The pellet was resuspended in 100 µl RIPA buffer (supplemented with Roche Complete Inhibitors 2×) and centrifuged again to remove any detergent-soluble proteins remaining. The final pellet containing detergent-insoluble proteins was solubilized in 75 µl 8 M Urea, 2% SDS, 50 mM DTT, 50 mM Tris pH 7.4 at room temperature.

For the total protein fraction, we solubilized 50 mg of the frozen animals from the sucrose separation directly into 225 µl 8 M Urea, 2% SDS, 50 mM DTT, 50 mM Tris pH 7.4. Levels of specific aggregating candidates were analyzed using the NuPAGE invitrogen gel system (Invitrogen, Carlsbad, CA, USA) with 4%–12% gradient gels. We chose six candidates for which antibodies were available and we detected bands of the appropriate size for the antibodies against 14-3-3 (Santa Cruz Biotechnology, Santa Cruz, CA, USA), RHO-1 (gift from Asako Sugimoto), HSP90 (Abcam, Cambridge, MA, USA), and casein kinase I-α (Cell Signaling, Danvers, MA, USA). Absolute intensity of each band was quantified using Photoshop (Adobe Photoshop CS) by subtracting the mean intensity from the background intensity and multiplied by the total pixels. Total protein levels were detected on the PVDF membrane, after transfer, by Sypro Ruby blot staining (Invitrogen, Carlsbad, CA, USA) following manufacturer's instructions. Fluorescence was detected using a Typhoon 9400 imager (GE Healthcare, Piscataway, NJ, USA) and quantified using Photoshop. To account for any differences in protein loading, we normalized the total levels of each protein detected by Western blot with whole lane protein levels measured by Sypro Ruby. As the overall levels of RIPA-insoluble proteins changed with age, we chose to normalize to four bands at ∼45, 90–95, and 200 kDa visible by Sypro Ruby that remained relatively constant with age. To estimate the fractional change in insolubility with age, we quantified the levels of protein in the insoluble fraction and divided this by the total protein levels for each candidate detected by Western blot, accounting for differences in extraction volumes and in quantities loaded on each gel. To determine the overall fold change with age in insoluble protein levels in *gon-2(−)* mutants subjected to control RNAi or *daf-2* RNAi, we quantified the Sypro Ruby staining between ∼45 and 80 kDa and below ∼45 kDa.

### Protein Digestion and iTRAQ Labeling

Dialyzed formic acid soluble proteins were resolubilized in 25 mM ammonium bicarbonate containing 6 M guanidine hydrochloride. Samples were further processed to reduce and alkylate cysteine side chains and digested with trypsin as described previously [24]. Peptides were labeled with iTRAQ following the manufacturer's instructions (Applied Biosystems, Pleasanton, CA, USA). We checked that over 95% of the peptides were labeled with iTRAQ by analyzing the sample on a 1-h LC-MS/MS run and searching the spectra, allowing iTRAQ as a variable modification.

### Strong Cation-Exchange Chromatography (SCX)

In order to identify as many peptides as possible, we performed extensive separation of the combined iTRAQ-labeled sample by SCX chromatography as described previously [24]. Briefly, we ran 90-min gradients from 0 to 350 mM KCl in 30% acetonitrile, 5 mM KH_2_PO_4_, pH 2.7 through a Tricorn 5/200 column (GE Healthcare, Piscataway, NJ, USA) packed in-house with 5 µm 300 Å polysulfoethyl A resin (Western Analytical, Lake Elsinore, CA, USA) using an ÄKTA Purifier (GE Healthcare, Piscataway, NJ, USA). We collected 60 fractions for further analysis and desalted these fractions using a MAX-RP reverse phase C_18_ cartridge (Phenomenex, Torrance, CA, USA). Varying the extent of SCX fractionation did not significantly increase the number of peptides identified. Desalted fractions were further diluted in 0.1% formic acid depending on their UV absorption.

### Nano-LC-ESI-Qq-TOF Tandem Mass Spectrometry Analysis

#### Method

Mass spectrometry analysis was performed as described previously [24]. Briefly, SCX fractions were further separated by reverse phase C_18_ column (LC Packings, Sunnyvale, CA, USA) with 90 min 3%–32% acetonitrile gradient cycles in 0.1% formic acid on an Agilent 1100 series HPLC (Agilent Technologies, Palo Alto, CA, USA). The LC eluent was coupled to a micro-ionspray source attached to a QSTAR Pulsar mass spectrometer (MDS Sciex, Foster City, CA, USA).

#### Data analysis

Data were visualized using Analyst QS software (version 1.1), and MS/MS centroid peak lists were generated using the Mascot.dll script (version 1.6b18). The MS/MS spectra were searched against the entire UniprotKB *C. elegans* database release 15.9 (downloaded 2009.10.13) using Protein Prospector v.5.3.2. Initial peptide tolerances in MS and MS/MS modes were 200 ppm and 0.2 daltons, respectively. Two missed cleavages were allowed for trypsin digestion. Carbamidomethylation and iTRAQ labeling of lysine residues were searched as fixed modifications. The peptide amino termini were fixed as either iTRAQ-modified or protein N-terminal acetylated. We allowed the following variable modifications: methionine oxidation, N-terminal acetylation, N-terminal acetylation and oxidation, N-terminal pyro-glutamate, and N-terminal methionine loss with or without acetylation. Other than N-terminal or methionine oxidation, we did not search specifically for oxidation of other amino acids, glycation, or nitrosylation. Therefore it is possible that we are missing some damaged peptides in our analysis, but the detection of the non-modified peptide as well as other peptides from the same protein should allow us to identify the majority of potentially damaged proteins. All high scoring peptide matches (expectation value<0.01) from individual LC-MS/MS runs were then used to internally recalibrate MS parent ion *m*/*z* values within that run. Recalibrated data files were then searched with a peptide tolerance in MS mode of 50 ppm. For each peptide MS/MS spectra, the raw area of the peaks at m/z 114.1, 115.1, 116.1, and 117.1 (±0.1 m/z) was determined by Protein Prospector from the raw data files. We kept only peptides that had at least one iTRAQ peak area over 25 counts. We performed a stringent quality control of the proteomic data by eliminating all proteins identified by one single peptide with an expectation value higher than 1E-3. For proteins identified by two or more peptides, we discarded peptides with an expectation value higher than 1E-2. The false positive rates were estimated by conducting the search using a concatenated database containing the original UniProt database as well as a version of each original entry where the sequence has been randomized. Using this stringent cut-off, we did not identify any proteins in the decoy-randomized database. For our list of aggregation-prone proteins, we kept only proteins identified in both experiments. In this list, we examined the MS/MS spectra manually for every protein quantified by a single iTRAQ-labeled peptide. Of these 38 proteins, 14 were discarded either because of poor quality spectra or due to the presence of a peptide other than the one of interest that was fragmented simultaneously. To determine the relative level of each protein in young or aged animals, we averaged the ratio of (peak area in young)/(peak area in young+peak area in aged) and, respectively, the ratio of (peak area in aged)/(peak area in young+peak area in aged) for all peptides identified for each protein. To measure the fold change in levels of aggregation-prone proteins with age, we divided the ratio for aged-animal proteins by the ratio for young-animal proteins.

### Bioinformatics Analysis

The *C. elegans* proteome set was prepared by obtaining all proteins identified by mass spectrometry available in PeptideAtlas (http://www.peptideatlas.org) with a PeptideProphet probability over 0.9. Proteins identified in our experiment but not in the PeptideAtlas database were added to this proteome set (final total: 7,826 proteins). To exclude possible biases in sequence or structural properties due to the presence of transmembrane helices or signal peptides, we removed all proteins predicted to contain transmembrane helices or signal peptides using TMHMM v2.0 [94] and SignalP v3.0 [95], respectively. To prevent bias due to redundancy, we reduced the sequence identity of the set to 50% using CD-HIT v3.1.1 [96], resulting in 5,094 proteins. These procedures were also applied to our aggregation-prone set, resulting in 530 proteins. Aliphatic amino acid residues were defined as A,G,I,L,V. Secondary structure content was predicted using PSIPRED v2.6 [67], and fold classifications were obtained from CATH v3.2 [68]. *p* values were calculated using the unequal variance *t* test.

Functional annotation of the aggregation-prone set was done using the freely available DAVID software (http://david.abcc.ncifcrf.gov/). The *C. elegans* proteome set of mass spectrometry detectable proteins was chosen as the background list. All gene ontology biological process terms were used for the analysis. Redundant categories were removed using the functional annotation clustering option.

To examine the overlap between proteins identified in human disease aggregates or insoluble proteins identified in the ALS mouse model and those in the *C. elegans* insoluble protein set, we identified the *C. elegans* orthologs using the Inparanoid eukaryotic ortholog database version 7.0 [97]. For human or mouse proteins not found in the Inparanoid database, we used BLAST to identify the closest homolog in *C. elegans* with an E-value<1E-5. The significance of overlap between the set of aggregation-prone proteins identified in this study and each set of insoluble proteins was calculated using the cumulative hypergeometric test.

### FRAP Analysis

Animals were anesthetized on agarose pads containing levamisole. FRAP analysis was performed using the Nikon C1si spectral confocal microscope (Nikon Center, UCSF). tagRFP and GFP fluorescence were detected using 561 nm and 488 nm lasers, respectively. Objective used: Plan Apo VC 100×/1.40 Oil (Nikon, Melville, NY, USA). Gain was kept between 6.5 and 8.5. Relative fluorescence intensity (RFI) was analyzed as described previously following the equation RFI = (T_t_/C_t_)/(T_0_/C_0_), where T_0_ is the intensity in the region of interest (ROI) before photobleaching; T_t_, the intensity in the ROI at a defined time after photobleaching; C_0_, the intensity in the non-bleached part of the puncta before photobleaching; and C_t_, the intensity in the non-bleached part of the puncta after bleaching [37]. Half-life recovery was determined using the GraphPad Prism 5 software by fitting the recovery curves with a least-squares regression.

### Western Blot Analysis of KIN-19 Expression Levels in Transgenic Animals

A standard SDS-PAGE protocol was followed. 100 N2 and 100 *pkin-19::kin-19::tagrfp* transgenic animals were collected, washed once in ddH_2_O, resuspended in NuPAGE loading buffer (Invitrogen, Carlsbad, CA, USA), and boiled. The equivalent of 20 animals was loaded on a 4%–12% gradient gel. The membrane was probed with anti-casein kinase I-α (Cell Signaling, Danvers, MA, USA) to detect endogenous and fluorescently tagged KIN-19.

### Relative Fluorescence Quantification

Animals were anesthetized on agarose pads in levamisole. Whole-worm images were taken using a Retiga EXi Fast1394 CCD digital camera (QImaging, Burnaby, British Columbia, Canada) using the 10× objective on a Zeiss Axioplan 2 compound microscope (Zeiss, Germany). Fluorescence quantification was performed using the Openlab 4.0.2 software by measuring the intensity of each pixel in the anterior pharyngeal bulb.

### RNAi Treatment

RNAi by feeding was performed as described previously [59]. The *kin-19* RNAi clone was obtained from the Marc Vidal library and sequenced. We began *kin-19* RNAi treatment at the last larval stage (L4) to avoid any developmental defects. Bacteria containing the empty vector L4440 were used as control.

### Aggregation Counting

Numbers of animals with less than 10, between 10 and 100, or more than 100 KIN-19::tagRFP puncta in the anterior pharyngeal bulb were determined using a Leica MZ16FA microscope (Leica, Bannockburn, IL, USA). *pkin-19::kin-19::tagrfp* in wild-type and *daf-2(−)* backgrounds were maintained at 15°C until the L4 stage and selected at this stage to avoid differences in developmental timing. Otherwise, animals were kept at 20°C. Q35 aggregate numbers were evaluated from photos taken of adult animals at Day 3 visualized with a Leica MZ16FA microscope. Day 3 is this first time point when more than a few aggregates could be observed in the transgenic animals. All counting was done in a blind fashion in which the identity of the samples was concealed.

### Paralysis Assay

Animals were kept at 20°C. *punc-54::q35::yfp; pkin-19::kin-19::tagrfp*, and *punc-54::q35::yfp; pkin-19::tagrfp* animals were selected at L4 stage. Animals were scored as paralyzed if they could move only their heads when poked with a platinum wire. The assay was done in a blind fashion in which the identity of the samples was concealed.

### Lifespan Analysis

Lifespan analysis was performed at 20°C as previously described 98. Stata 8.2 (SAS) software was used for statistical analysis. *p* values were calculated using the Log-rank (Mantel-Cox) method.

### Statistics

Statistics were performed using Excel and GraphPad Prism 5. Cumulative hypergeometric probabilities were calculated using the free online Stat Trek software (http://stattrek.com) or MATLAB and using the proteome detectable by mass spectrometry as the population size (total: 7,826 proteins).

## Supporting Information

Figure S1 **Flowchart describing the insoluble-protein extraction procedure and overlap between experiments.** (A) Flowchart of the sequential extraction to isolate aggregation-prone proteins (detailed in the Methods section). The insoluble fraction is expected to contain aggregated proteins and insoluble but functional proteins. (B) Venn diagram showing that we identified 1,125 proteins in Experiment 1 and 856 in Experiment 2, of which 725 proteins were identified in both experiments (see text). The cumulative hypergeometric probability of observing an overlap of 725 proteins is less than 1E-100. These results show that we could reproducibly identify the large majority of proteins in the insoluble fraction.
(0.11 MB PDF)

Figure S2 **Flowchart describing the proteomic experiment with iTRAQ quantification.** Flowchart of procedures from extraction to mass spectrometry analysis and quantification. The experiment was carried out twice: Experiment 1 with *fem-1(−)* animals and gonad-less [*gon-2(−)*] animals and Experiment 2 with *fem-1(−)* animals and germline-less [*glp-1(−)*] animals. *fem-1(−)* animals lack sperm but contain both somatic and germline tissue including oocytes.
(0.01 MB PDF)

Figure S3 **Expression of KIN-19::tagRFP in transgenic animals.** (A) KIN-19::tagRFP expression pattern in young *Pkin-19::kin-19::tagrfp* animal. KIN-19::tagRFP was highly expressed in the pharynx and dorsal, ventral, and lateral neuronal processes. The right panel shows a Nomarski photograph of the same animal. (B) Western blot detection of endogenous and fluorescent-tagged KIN-19::tagRFP with anti-casein kinase I-α antibody in young adults. Quantification of endogenous and fluorescent-tagged KIN-19::tagRFP bands with ImageJ (NIH) showed similar mean values for both bands (integrated density: KIN-19::tagRFP, 2.4; KIN-19, 2.5). This suggests an equivalent expression of the transgene and endogenous proteins. (C) Lifespan analysis of *Pkin-19::kin-19::tagrfp* and N2 animals at 20°C. *Pkin-19::kin-19::tagrfp* animals had a mean lifespan of 18.4 days (59 events observed/82 total) and N2, 20 days (82 events observed/96 total). Transgenic compared to control: *p* = 0.085. (D) Time-course of KIN-19::tagRFP aggregation in a population of *Pkin-19::kin-19::tagrfp* animals aging at 20°C. Animals were counted as containing KIN-19::tagRFP aggregates if over ten such aggregates were present in the anterior pharyngeal bulb. Experiment was started with 50 animals.
(0.05 MB PDF)

Figure S4 **KIN-19::tagRFP becomes immobile in aged BDU neuronal processes and reducing KIN-19 levels does not prevent aggregation in the pharynx.** (A) KIN-19::tagRFP became immobile with age in one lateral neuron (BDU). FRAP in *Pkin-19::kin-19::tagrfp* animals, Day 15 (Laser setting: 20% in 0.5 µm^2^). (B) In young animals, KIN-19::tagRFP was mobile in the BDU process, Day 3 (Laser setting: 40% in 0.76 µm^2^); Scale bar: 2 µm. (C) KIN-19::tagRFP puncta in animals subjected to *kin-19* RNAi contained immobile protein as measured by FRAP. *Pkin-19::kin-19::tagrfp* treated with *kin-19* RNAi, Day 6. No recovery in fluorescence was observed between 8 and 338 s after photo-bleaching. Laser setting: 20% in 0.5 µm^2^. Scale bar: 1 µm.
(0.05 MB PDF)

Figure S5 **Total protein levels are not correlated with age-dependent changes in protein insolubility.** (A) Total levels of aggregation-prone candidates in aged compared to young animals remained similar or decreased in older animals. Histogram represents the quantification of total protein levels that were detected by Western blot in two biological independent samples. Total protein level is represented as a percentage of total protein detected in young animals. Total DAF-21 levels were reduced on average by 1.47-fold with age. (B) Insolubility affects a small proportion of the total amount of aggregation-prone protein available. In aged animals, 35.9% of total proteins probed in *fem-(−)* and 10.4% of total proteins probed by Western blot in *gon-2(−)* animals are in an insoluble form. Overall, insolubility affects a higher proportion of total protein in the animals containing reproductive tissues versus only somatic tissues. It remains unclear why RHO-1 does not follow this trend (Figure 3), although we clearly show its aggregation in oocytes. One explanation could be the high variability of the detection of RHO-1 insolubility in aged *gon-2(−)* animals by Western blot (3.9% and 16.7% of total protein in independent experiments). (C) With age, DAF-21/HSP90 becomes truncated and highly insoluble. Western blot detection of DAF-21/HSP90 in soluble (in RIPA buffer) and insoluble (pellet in Urea and SDS buffer) fractions from animals with only somatic tissues [*gon-2(−)*]. Full arrowhead indicates full-length DAF-21/HSP90. An open arrowhead shows urea-insoluble DAF-21/HSP90 localized in the gel well detected in aged animals and an asterisk points to a 17 kDa cleavage product formed both in the soluble and insoluble fractions in aged animals.
(0.06 MB PDF)

Figure S6 **Over-expression of the aggregation-prone protein KIN-19 is correlated with a small decrease in Q35 aggregates.** Animals with muscle-aggregated KIN-19 have slightly fewer large Q35 aggregates. Low magnification visible Q35 aggregates were counted blind in *Punc-54::q35::yfp; Pmyo-3::tagrfp* animals and *Punc-54::q35::yfp*; *Pmyo-3::kin-19::tagrfp*. Number of animals evaluated shown on *x*-axis. Day 3. Error bars: SEM. Kruskal-Wallis test, *p*<0.0001.
(0.01 MB PDF)

Figure S7 **Bioinformatic analysis of structural similarities among aggregation-prone proteins.** (A–D) Bioinformatic analysis of aggregation-prone proteins (red) compared to the *C. elegans* proteome compiled from proteins detected by mass spectrometry (black). (A) We found a significant change in amino acid composition in aggregation-prone proteins compared to the proteome. Statistical significance was determined as a difference greater than 0.3%, * *p*<0.001 between amino acid composition in the insoluble set versus the proteome. Alanine (A), glycine (G), and valine (V) were significantly over-represented (unequal variance *t* test: A, *p* = 2.4E-26; G, *p* = 1.7E-26 and V, *p* = 1.9E-18) and cysteine (C), phenylalanine (F), methionine (M), asparagine (N), proline (P), and serine (S) were significantly under-represented in the aggregation-prone set (unequal variance *t* test: C, *p* = 1.8E-11; F, *p* = 8.7E-7; M, *p* = 5.4E-15; N, *p* = 5.8E-9; P, *p* = 1.3E-8, and S, *p* = 1.6E-26). (B) We found a significant increase in predicted β-sheets content in aggregation-prone proteins (unequal variance *t* test, *p* = 6.2E-6). We note that the difference in β-sheets content is distributed throughout the range, suggesting that this enrichment is not caused by a small class of proteins. (C) We found no significant difference in levels of α-helical content (unequal variance *t* test, *p* = 0.35). (D) Our aggregation-prone protein set was enriched in proteins with mixed α-helix and β-sheet folds (in particular 3.40 and 3.50 CATH folds) and proteins with β-sheet folds (in particular 2.40 CATH fold) but contains fewer proteins with α-helix rich (in particular 1.10 CATH folds). The number of proteins per fold observed in the aggregation-prone protein set and the respective number of proteins expected in the proteome are shown (only folds identified in at least 12 proteins are displayed). Significance was evaluated by chi-test comparing all folds identified in the aggregation-prone protein set to expected numbers in the proteome: *p* = 1.9E-5.
(0.09 MB PDF)

Figure S8 **Lifespan analysis of strains used. Lifespan analysis of sterile mutants compared to N2 animals at 25°C.** All sterile mutants used for the proteomic study had a significantly longer lifespan than control when kept at 25°C. Lifespan of *fem-1(hc17)*: mean = 14.4, *n* = 101 (observed)/104(total), *p* versus control = 0.002; *glp-1(e2141)*: *m* = 13.7, *n* = 103/107, *p* versus control = 0.01; *gon-2(q388)*: *m* = 15.8, *n* = 95/100, *p* versus control<0.0001; control N2: *m* = 11.9, *n* = 73/94. We found no significant differences in lifespan between sterile mutants at 25°C.
(0.01 MB PDF)

Table S1 **Insoluble proteins identified in** ***C. elegans*** **.** Grey highlight marks the set of 461 proteins that consistently became 1.5-fold or more insoluble with age in all four datasets.
(1.27 MB XLS)

Table S2 **Cytoskeletal proteins identified in the insoluble fraction.**
(0.07 MB XLS)

Table S3 **Age-dependent aggregation-prone proteins identified as significantly regulated in** ***daf-2(e1370)*** **versus** ***daf-16(mu86)*** **;** ***daf-2(e1370)*** **microarrays (Shaw et al. 2007** [**47**] **).** Microarray data from Shaw et al. comparing *daf-2(e1370)* versus *daf-16(mu86)*; *daf-2(e1370)* identified 1,570 genes up-regulated and 796 down-regulated in daf-2 mutants (*q*<0.1, 17,965 genes in total). The mRNA levels of 457 age-dependent aggregation-prone proteins were evaluated in these microarrays. We note that mRNA levels of *rho-1* were down-regulated in *daf-2(−)* mutants, but the protein levels of RHO-1 remained unchanged as detected by Western blot possibly reflecting the variation between mRNA and protein levels as previously described in Gygi et al. (1999) Mol Cell Biol 19: 1720–1730.
(0.10 MB XLS)

Table S4 **Paralysis of** ***C. elegans*** **expressing muscle polyglutamine Q35 with or without muscle KIN-19.** The assay was done in a blind fashion in which the identity of the samples was concealed. (*): Animals were maintained at 15°C until L4 stage and then transferred to 20°C. All other experiments were continuously kept at 20°C. (#): Between the times we performed experiments 3 and 6, we carried out two experiments that showed no difference in paralysis between the experimental and control animals. We noted that the paralysis in the control animals was higher than average, which could explain why we saw no difference. However it remains unclear which experimental variable could account for these results. Furthermore, variability in the phenotype of polyglutamine-repeat transgenics has been previously reported [19].
(0.05 MB PDF)

Table S5 **(A) Specific functional categories are over-represented in the age-dependent insoluble protein set.** Functional annotation was carried out using the DAVID software. A total of 450 out of 461 age-dependent insoluble proteins were recognized by DAVID and 349 of these fell into one or more significant gene ontology biological process category. EASE score *p* value: modified Fisher exact *p* value. (B) Specific functional categories are over-represented in the age-independent insoluble protein set. Functional annotation was carried out using the DAVID software. A total of 243 out of 250 age-independent insoluble proteins were recognized by DAVID and 214 of these fell into one or more significant gene ontology biological process category. EASE score *p* value: modified Fisher exact *p* value.
(0.02 MB PDF)

Table S6 **Proteins identified as aggregation-prone and identified in RNAi screens for extended lifespan.** 27 out of 29 genes identified in Hansen et al. (2005) [59] and 56 out of 64 genes identified in Curran et al. (2007) [58] were present in our compiled mass spectrometry proteome. Grey highlight marks proteins found in the age-dependent insoluble set.
(0.08 MB XLS)

Table S7 **Primary and predicted secondary structures identified in aggregation-prone proteins.** *p* values were calculated using the unequal variance *t* test.
(0.03 MB XLS)

Table S8 **Proteins identified as aggregation-prone and identified in RNAi screens for increased polyglutamine aggregation (Nollen et al., 2004** [**20**] **).** 156 out of 186 genes identified in Nollen et al. (2004) were present in our compiled mass spectrometry proteome dataset. Grey highlight marks proteins found in the age-dependent insoluble set. (*) Also found to increase lifespan when inhibited by RNAi.
(0.14 MB XLS)

Table S9 **Amyloid plaque components identified as aggregation-prone in** ***C. elegans*** **.** (#) Proteomic characterization of postmortem amyloid plaques isolated by laser capture microdissection (Liao L et al., J Biol Chem. 2004 Aug 27; 279 (35):37061–8 [16]). Organism: human. (*) identified with BLAST (closest homolog with e-value<1-e05). Cumulative hypergeometric test *p*(X≥13) = 2.5E-8.
(0.03 MB XLS)

Table S10 **Neurofibrillary tangle components identified as aggregation-prone in** ***C. elegans*** **.** (#) Proteomic analysis of neurofibrillary tangles in Alzheimer disease identifies GAPDH as a detergent-insoluble paired helical filament tau binding protein (Wang, Q et al., Faseb J. 2005 May;19(7):869–71 [18]). Organism: human. (*) identified with BLAST (closest homolog with e-value<1-e05). Cumulative hypergeometric test *p*(X≥32)≈0.
(0.03 MB XLS)

Table S11 **Lewy body components identified as aggregation-prone in** ***C. elegans*** **.** (#) Proteomic identification of novel proteins associated with Lewy bodies (Xia Q et al., Front Biosci. 2008 May 1;13:3850–6 [17]). Organism: Human. (*) identified with BLAST (closest homolog with e-value<1-e05). Cumulative hypergeometric test *p*(X≥11) = 0.0002.
(0.05 MB XLS)

Table S12 **Detergent-insoluble proteins identified in mouse model for ALS and in** ***C. elegans*** **.** (#) Characterization of detergent-insoluble proteins in ALS indicates a causal link between nitrative stress and aggregation in pathogenesis (Basso M et al., PLoS One. 2009 Dec 2; 4(12):e8130 [93]). Organism: mouse. (*) identified with BLAST (closest homolog with e-value<1-e05). Cumulative hypergeometric test *p*(X≥22)≈0.
(0.05 MB XLS)

## Abbreviations

ALS: amyotrophic lateral sclerosis
FRAP: fluorescent recovery after photobleaching
sIBM: sporadic inclusion body myositis
