# Supplementary figures and images for: Widespread Protein Aggregation as an Inherent Part of Aging in C. elegans
Source: PLoS Biol. 2010 Aug 10;8(8):e1000450. doi: 10.1371/journal.pbio.1000450 (PMC2919420; doi:10.1371/journal.pbio.1000450)

Figure S1

A

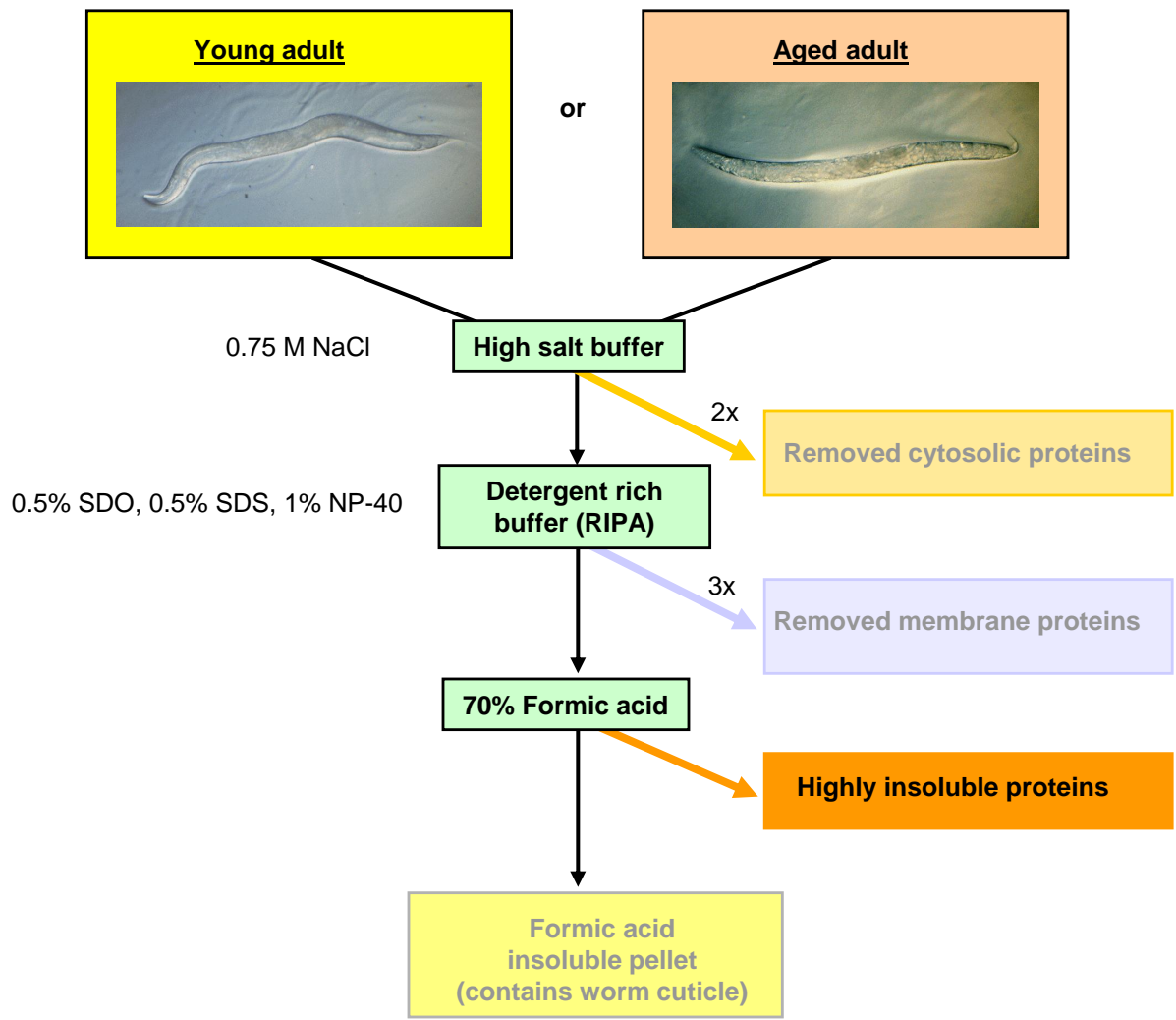

B

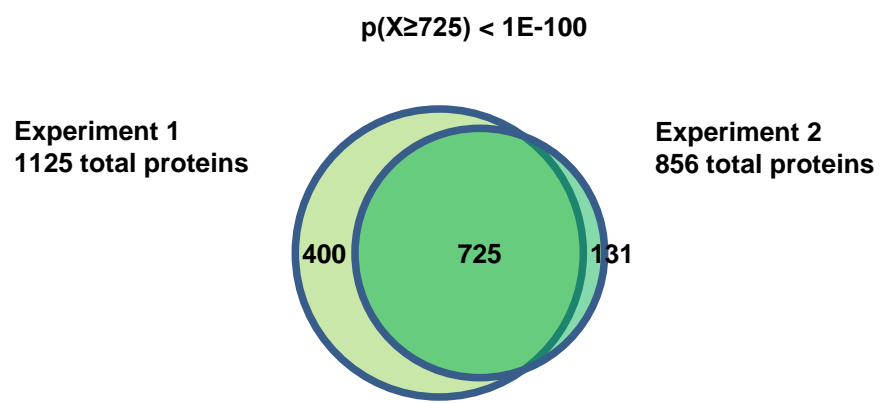

Supplement: Figure S1 — Flowchart describing the insoluble-protein extraction procedure and overlap between experiments. (A) Flowchart of the sequential extraction to isolate aggregation-prone proteins (detailed in the Methods section). The insoluble fraction is expected to contain aggregated proteins and insoluble but functional proteins. (B) Venn diagram showing that we identified 1,125 proteins in Experiment 1 and 856 in Experiment 2, of which 725 proteins were identified in both experiments (see text). The cumulative hypergeometric probability of observing an overlap of 725 proteins is less than 1E-100. These results show that we could reproducibly identify the large majority of proteins in the insoluble fraction. (0.11 MB PDF) [file pbio.1000450.s001.pdf]

**Figure S2**

**Experiment 1**

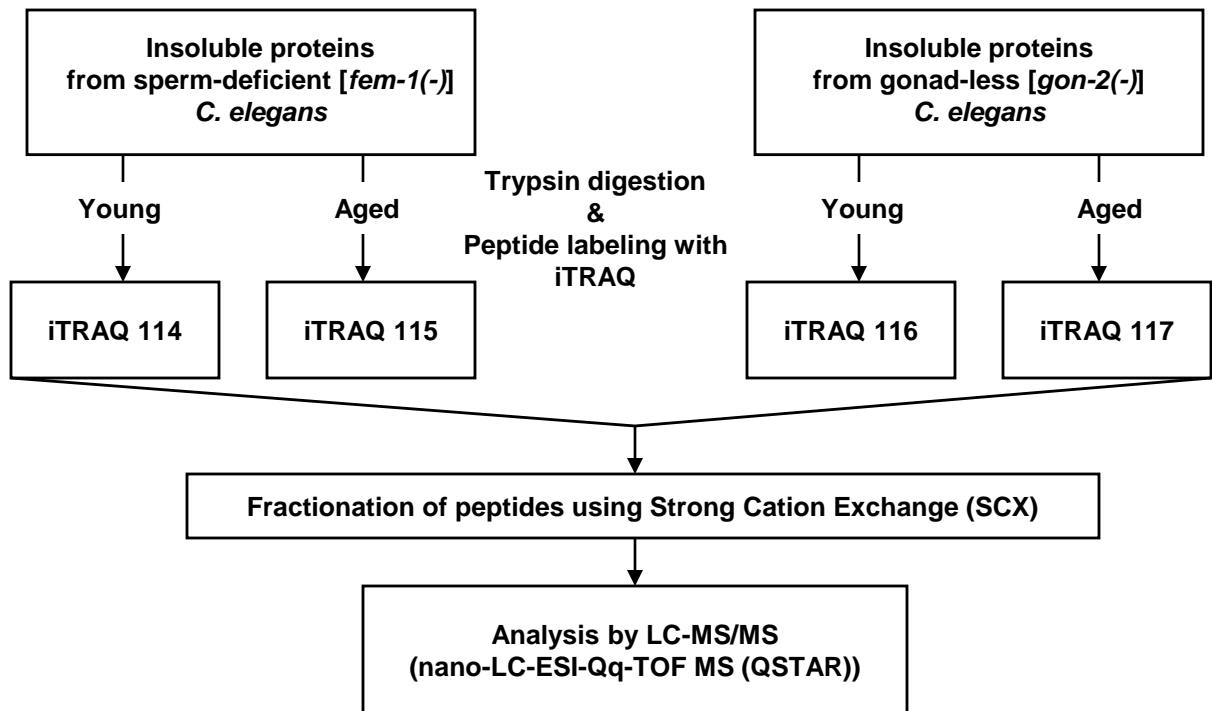

**Experiment 2**

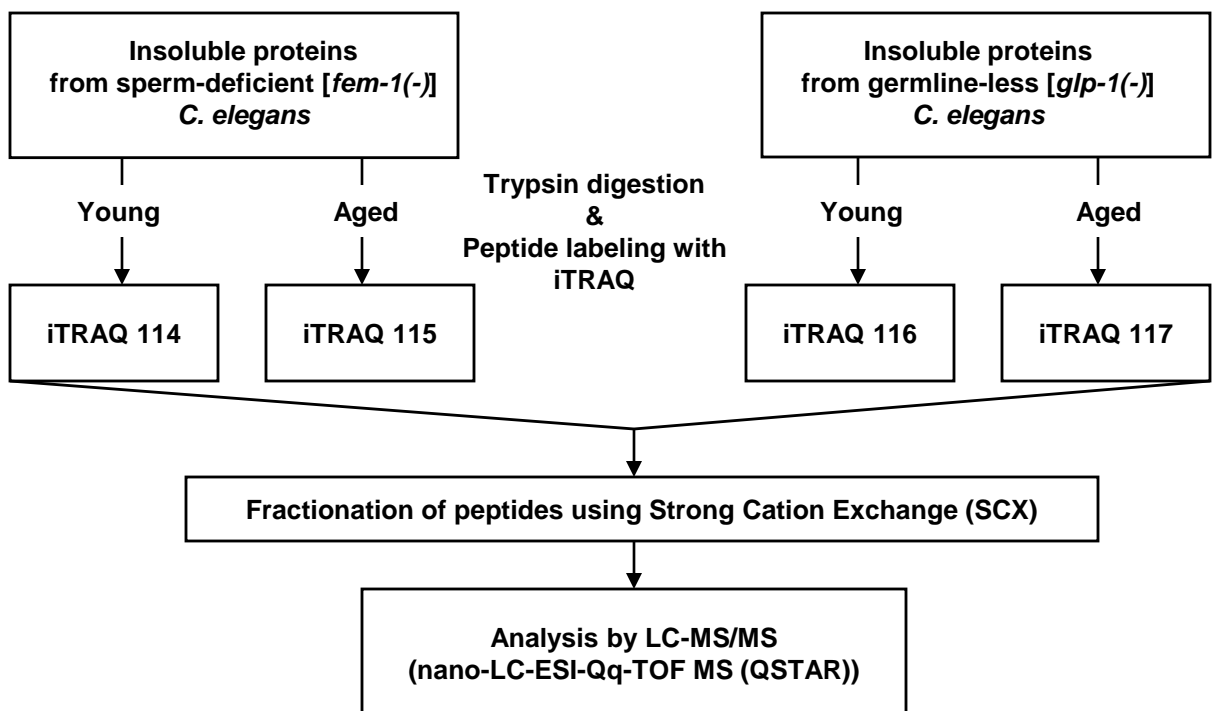

Supplement: Figure S2 — Flowchart describing the proteomic experiment with iTRAQ quantification. Flowchart of procedures from extraction to mass spectrometry analysis and quantification. The experiment was carried out twice: Experiment 1 with fem-1(−) animals and gonad-less [gon-2(−)] animals and Experiment 2 with fem-1(−) animals and germline-less [glp-1(−)] animals. fem-1(−) animals lack sperm but contain both somatic and germline tissue including oocytes. (0.01 MB PDF) [file pbio.1000450.s002.pdf]

Figure S3

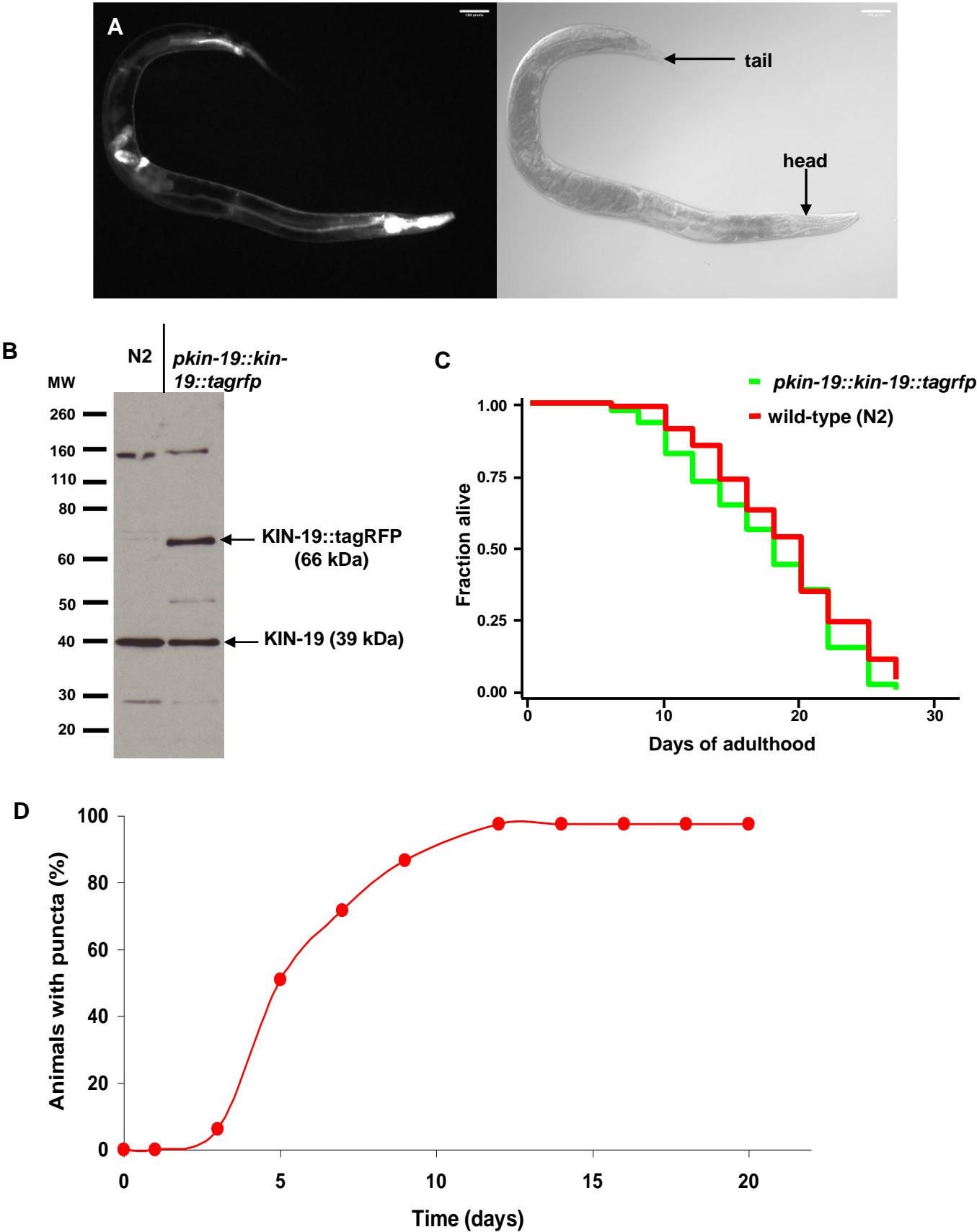

Supplement: Figure S3 — Expression of KIN-19::tagRFP in transgenic animals. (A) KIN-19::tagRFP expression pattern in young Pkin-19::kin-19::tagrfp animal. KIN-19::tagRFP was highly expressed in the pharynx and dorsal, ventral, and lateral neuronal processes. The right panel shows a Nomarski photograph of the same animal. (B) Western blot detection of endogenous and fluorescent-tagged KIN-19::tagRFP with anti-casein kinase I-α antibody in young adults. Quantification of endogenous and fluorescent-tagged KIN-19::tagRFP bands with ImageJ (NIH) showed similar mean values for both bands (integrated density: KIN-19::tagRFP, 2.4; KIN-19, 2.5). This suggests an equivalent expression of the transgene and endogenous proteins. (C) Lifespan analysis of Pkin-19::kin-19::tagrfp and N2 animals at 20°C. Pkin-19::kin-19::tagrfp animals had a mean lifespan of 18.4 days (59 events observed/82 total) and N2, 20 days (82 events observed/96 total). Transgenic compared to control: p = 0.085. (D) Time-course of KIN-19::tagRFP aggregation in a population of Pkin-19::kin-19::tagrfp animals aging at 20°C. Animals were counted as containing KIN-19::tagRFP aggregates if over ten such aggregates were present in the anterior pharyngeal bulb. Experiment was started with 50 animals. (0.05 MB PDF) [file pbio.1000450.s003.pdf]

Figure S5

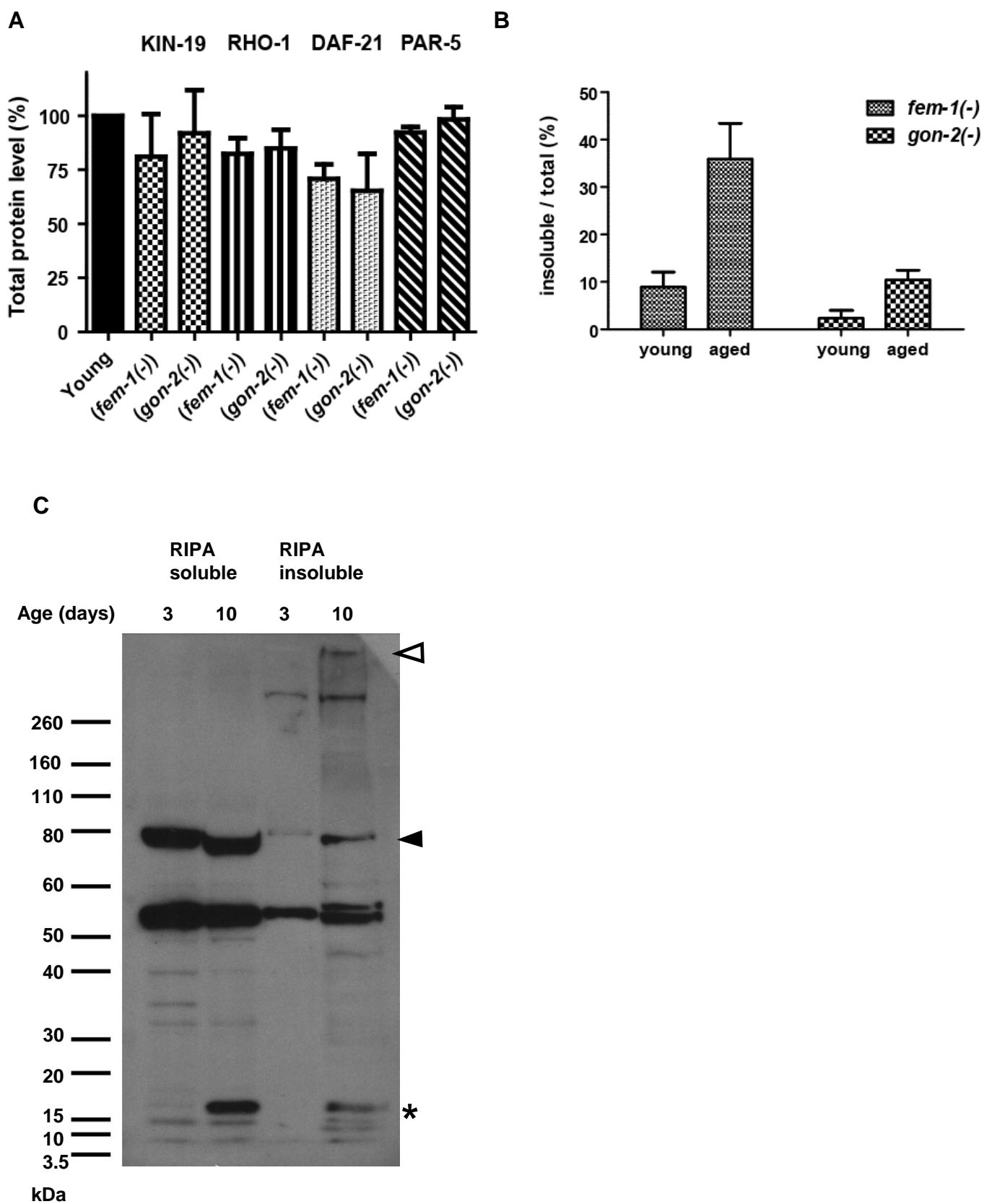

Supplement: Figure S5 — Total protein levels are not correlated with age-dependent changes in protein insolubility. (A) Total levels of aggregation-prone candidates in aged compared to young animals remained similar or decreased in older animals. Histogram represents the quantification of total protein levels that were detected by Western blot in two biological independent samples. Total protein level is represented as a percentage of total protein detected in young animals. Total DAF-21 levels were reduced on average by 1.47-fold with age. (B) Insolubility affects a small proportion of the total amount of aggregation-prone protein available. In aged animals, 35.9% of total proteins probed in fem-(−) and 10.4% of total proteins probed by Western blot in gon-2(−) animals are in an insoluble form. Overall, insolubility affects a higher proportion of total protein in the animals containing reproductive tissues versus only somatic tissues. It remains unclear why RHO-1 does not follow this trend (Figure 3), although we clearly show its aggregation in oocytes. One explanation could be the high variability of the detection of RHO-1 insolubility in aged gon-2(−) animals by Western blot (3.9% and 16.7% of total protein in independent experiments). (C) With age, DAF-21/HSP90 becomes truncated and highly insoluble. Western blot detection of DAF-21/HSP90 in soluble (in RIPA buffer) and insoluble (pellet in Urea and SDS buffer) fractions from animals with only somatic tissues [gon-2(−)]. Full arrowhead indicates full-length DAF-21/HSP90. An open arrowhead shows urea-insoluble DAF-21/HSP90 localized in the gel well detected in aged animals and an asterisk points to a 17 kDa cleavage product formed both in the soluble and insoluble fractions in aged animals. (0.06 MB PDF) [file pbio.1000450.s005.pdf]

Figure S6

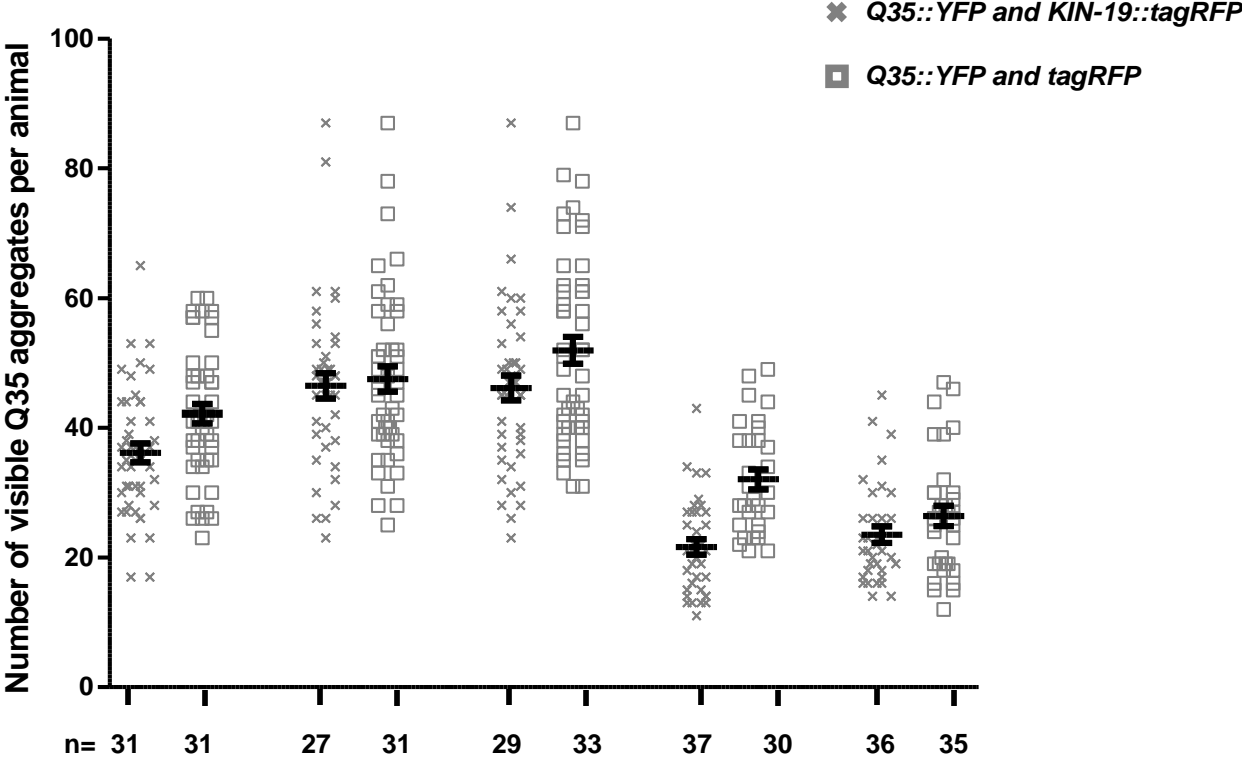

Supplement: Figure S6 — Over-expression of the aggregation-prone protein KIN-19 is correlated with a small decrease in Q35 aggregates. Animals with muscle-aggregated KIN-19 have slightly fewer large Q35 aggregates. Low magnification visible Q35 aggregates were counted blind in Punc-54::q35::yfp; Pmyo-3::tagrfp animals and Punc-54::q35::yfp; Pmyo-3::kin-19::tagrfp. Number of animals evaluated shown on x-axis. Day 3. Error bars: SEM. Kruskal-Wallis test, p<0.0001. (0.01 MB PDF) [file pbio.1000450.s006.pdf]

Figure S7

Aggregation-prone protein set  
Proteome

A

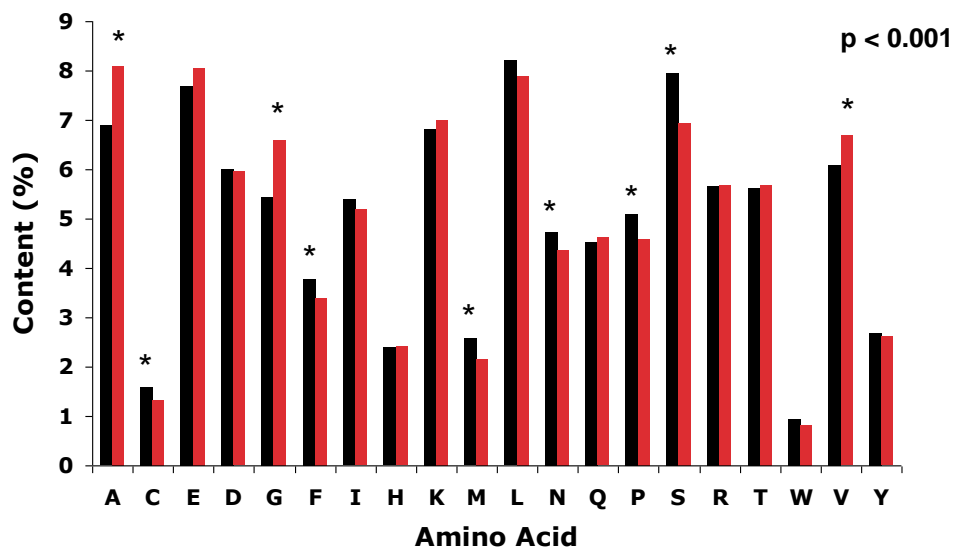

B

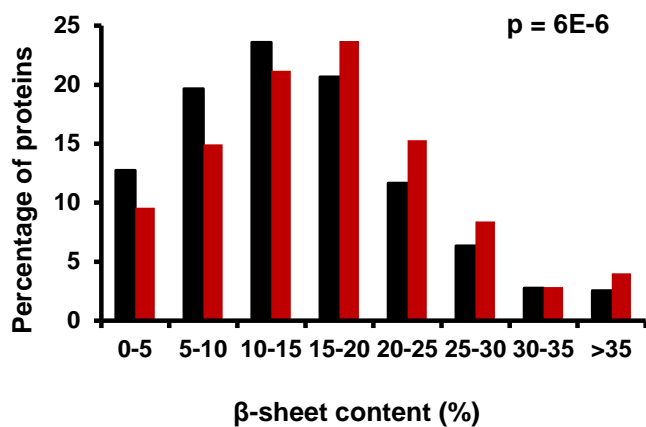

C

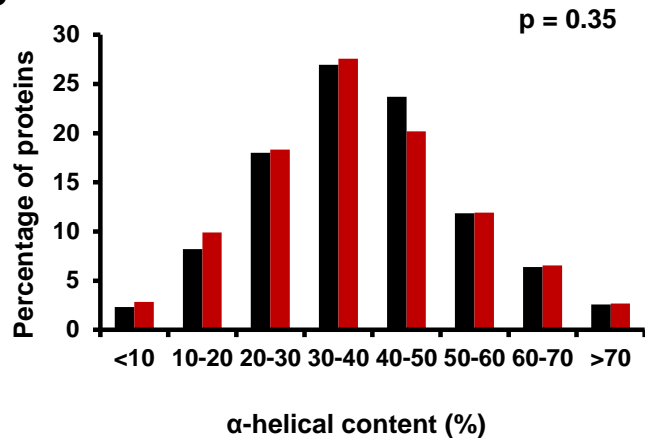

D

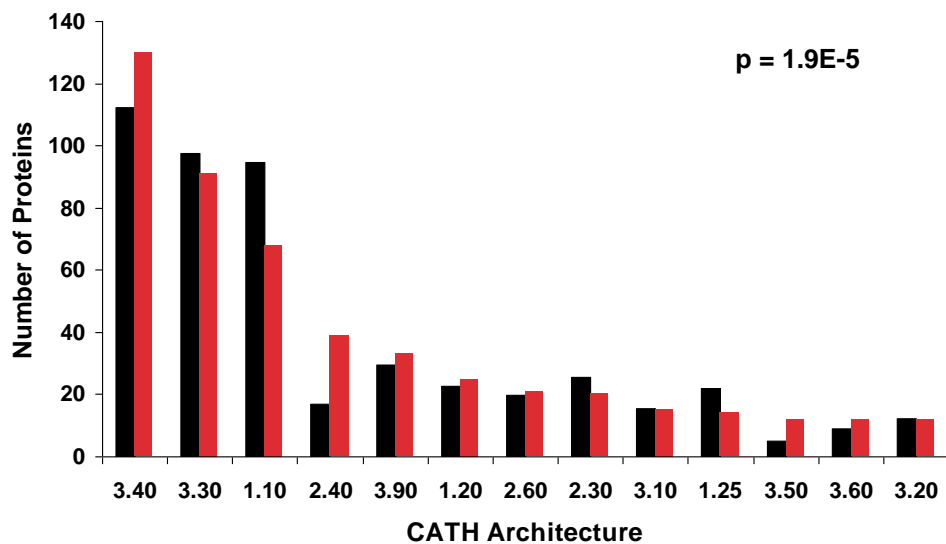

Supplement: Figure S7 — Bioinformatic analysis of structural similarities among aggregation-prone proteins. (A–D) Bioinformatic analysis of aggregation-prone proteins (red) compared to the C. elegans proteome compiled from proteins detected by mass spectrometry (black). (A) We found a significant change in amino acid composition in aggregation-prone proteins compared to the proteome. Statistical significance was determined as a difference greater than 0.3%, * p<0.001 between amino acid composition in the insoluble set versus the proteome. Alanine (A), glycine (G), and valine (V) were significantly over-represented (unequal variance t test: A, p = 2.4E-26; G, p = 1.7E-26 and V, p = 1.9E-18) and cysteine (C), phenylalanine (F), methionine (M), asparagine (N), proline (P), and serine (S) were significantly under-represented in the aggregation-prone set (unequal variance t test: C, p = 1.8E-11; F, p = 8.7E-7; M, p = 5.4E-15; N, p = 5.8E-9; P, p = 1.3E-8, and S, p = 1.6E-26). (B) We found a significant increase in predicted β-sheets content in aggregation-prone proteins (unequal variance t test, p = 6.2E-6). We note that the difference in β-sheets content is distributed throughout the range, suggesting that this enrichment is not caused by a small class of proteins. (C) We found no significant difference in levels of α-helical content (unequal variance t test, p = 0.35). (D) Our aggregation-prone protein set was enriched in proteins with mixed α-helix and β-sheet folds (in particular 3.40 and 3.50 CATH folds) and proteins with β-sheet folds (in particular 2.40 CATH fold) but contains fewer proteins with α-helix rich (in particular 1.10 CATH folds). The number of proteins per fold observed in the aggregation-prone protein set and the respective number of proteins expected in the proteome are shown (only folds identified in at least 12 proteins are displayed). Significance was evaluated by chi-test comparing all folds identified in the aggregation-prone protein set to expected numbers in the prot [file pbio.1000450.s007.pdf]

Figure S8

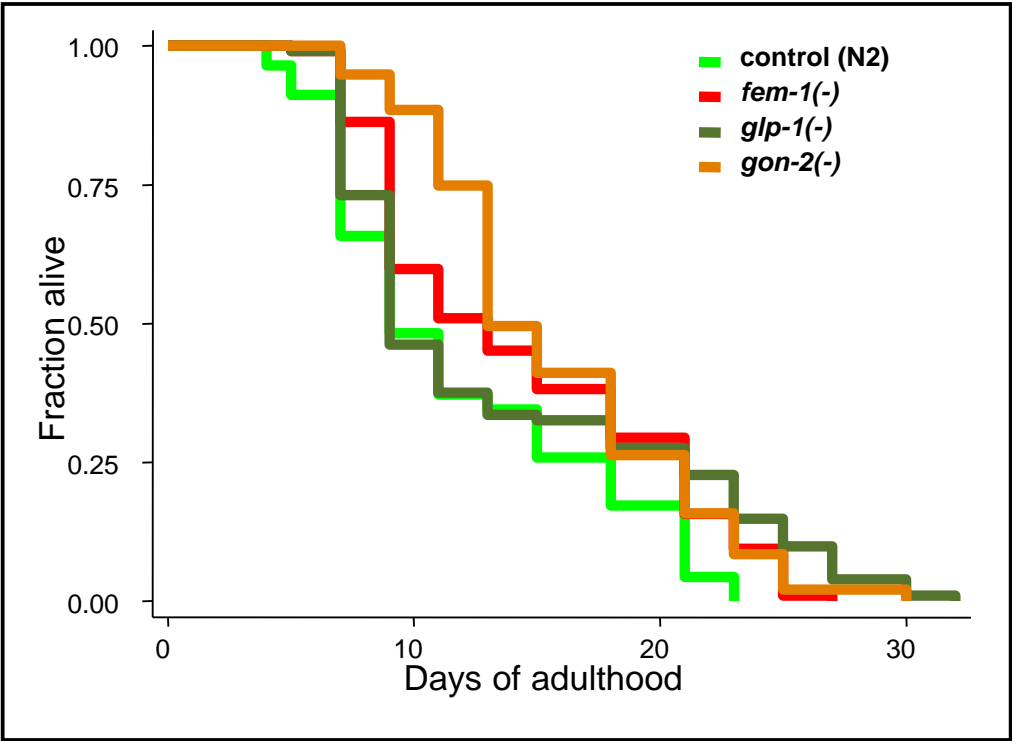

Supplement: Figure S8 — Lifespan analysis of strains used. Lifespan analysis of sterile mutants compared to N2 animals at 25°C. All sterile mutants used for the proteomic study had a significantly longer lifespan than control when kept at 25°C. Lifespan of fem-1(hc17): mean = 14.4, n = 101 (observed)/104(total), p versus control = 0.002; glp-1(e2141): m = 13.7, n = 103/107, p versus control = 0.01; gon-2(q388): m = 15.8, n = 95/100, p versus control<0.0001; control N2: m = 11.9, n = 73/94. We found no significant differences in lifespan between sterile mutants at 25°C. (0.01 MB PDF) [file pbio.1000450.s008.pdf]
