# Supplementary material for: Widespread Protein Aggregation as an Inherent Part of Aging in C. elegans
Source: PLoS Biol. 2010 Aug 10;8(8):e1000450. doi: 10.1371/journal.pbio.1000450 (PMC2919420; doi:10.1371/journal.pbio.1000450)
Supplement: Figure S4 — KIN-19::tagRFP becomes immobile in aged BDU neuronal processes and reducing KIN-19 levels does not prevent aggregation in the pharynx. (A) KIN-19::tagRFP became immobile with age in one lateral neuron (BDU). FRAP in Pkin-19::kin-19::tagrfp animals, Day 15 (Laser setting: 20% in 0.5 µm2). (B) In young animals, KIN-19::tagRFP was mobile in the BDU process, Day 3 (Laser setting: 40% in 0.76 µm2); Scale bar: 2 µm. (C) KIN-19::tagRFP puncta in animals subjected to kin-19 RNAi contained immobile protein as measured by FRAP. Pkin-19::kin-19::tagrfp treated with kin-19 RNAi, Day 6. No recovery in fluorescence was observed between 8 and 338 s after photo-bleaching. Laser setting: 20% in 0.5 µm2. Scale bar: 1 µm. (0.05 MB PDF) [file pbio.1000450.s004.pdf]

Figure S4

A

BDU process in KIN-19::tagRFP animal, day 15 (immobile)

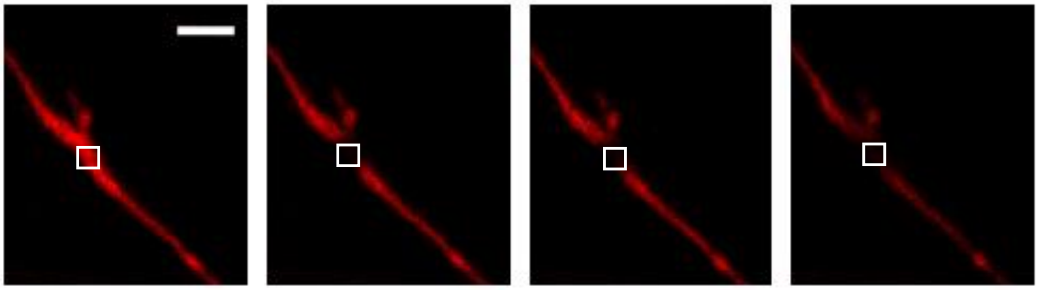

Before photo-bleaching | **After 8 sec** | **After 38 sec** | **After 278 sec**

B

BDU process in KIN-19::tagRFP animal, day 3 (mobile)

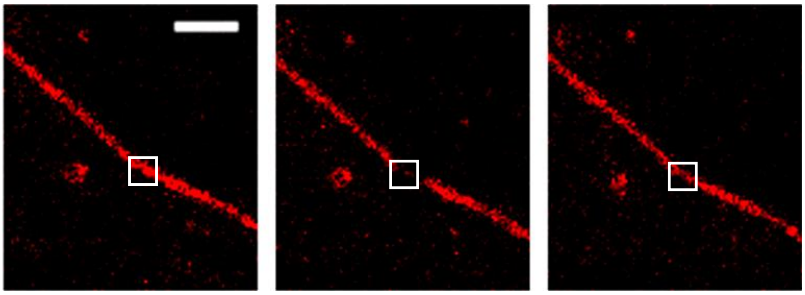

Before photo-bleaching | **After 8 sec** | **After 278 sec**

C

KIN-19::tagRFP expressing animals with *kin-19* RNAi, day 6

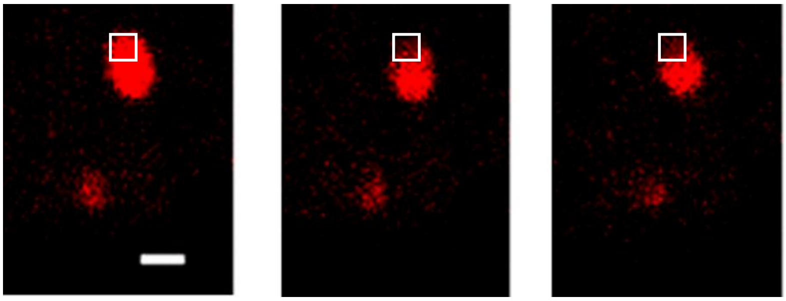

Before photo-bleaching | **After 8 sec** | **After 338 sec**
