# Supplementary material for: Widespread Protein Aggregation as an Inherent Part of Aging in C. elegans
Source: PLoS Biol. 2010 Aug 10;8(8):e1000450. doi: 10.1371/journal.pbio.1000450 (PMC2919420; doi:10.1371/journal.pbio.1000450)
Supplement: Table S4 — Paralysis of C. elegans expressing muscle polyglutamine Q35 with or without muscle KIN-19. The assay was done in a blind fashion in which the identity of the samples was concealed. (*): Animals were maintained at 15°C until L4 stage and then transferred to 20°C. All other experiments were continuously kept at 20°C. (#): Between the times we performed experiments 3 and 6, we carried out two experiments that showed no difference in paralysis between the experimental and control animals. We noted that the paralysis in the control animals was higher than average, which could explain why we saw no difference. However it remains unclear which experimental variable could account for these results. Furthermore, variability in the phenotype of polyglutamine-repeat transgenics has been previously reported [19]. (0.05 MB PDF) [file pbio.1000450.s012.pdf]

Table S4: Paralysis of *C. elegans* expressing muscle polyglutamine Q35 with or without muscle KIN-19

Experiment	Strain	Day 6			Day 7			Day 8		
		Number of animals (n)	% paralyzed	P-value	Number of animals (n)	% paralyzed	P-value	Number of animals (n)	% paralyzed	P-value
1	KIN-19-tagRFP + Q35	129	41.9		123	55.3		104	72.1	
	tagRFP + Q35	106	18.9	0.0003	96	35.4	0.005	90	62.2	0.19
2*	KIN-19-tagRFP + Q35	66	68.2		65	76.9		55	85.5	
	tagRFP + Q35	76	25.0	<0.0001	73	31.5	<0.0001	69	56.5	0.001
3	KIN-19-tagRFP + Q35	120	35.8		104	72.1		87	73.6	
	tagRFP + Q35	94	14.9	0.001	76	50.0	0.004	71	78.9	0.55
4#	KIN-19-tagRFP + Q35	104	51.9							
	tagRFP + Q35	61	37.7	0.108						
5#	KIN-19-tagRFP + Q35	98	33.7							
	tagRFP + Q35	94	33.0	0.9187						
6	KIN-19-tagRFP + Q35	143	18.9		134	55.2		130	87.7	
	tagRFP + Q35	123	1.6	<0.0001	113	21.2	<0.0001	107	63.6	0.13
	Q35 alone	142	0.7	<0.0001	129	27.1	<0.0001	122	82.8	0.83

The assay was done in a blind fashion in which the identity of the samples was concealed.

(*): Animals were maintained at 15°C until L4 stage and then transferred to 20°C. All other experiments were continuously kept at 20°C.

(#): Between the times we performed experiments 3 and 6, we carried out two experiments that showed no difference in paralysis between the experimental and control animals. We noted that the paralysis in the control animals was higher than average, which could explain why we saw no difference. However it remains unclear which experimental variable could account for these results. Furthermore, variability in the phenotype of polyglutamine-repeat transgenics has been previously reported (T. Gidalevitz, A. Ben-Zvi, K. H. Ho, H. R. Brignull, R. I. Morimoto, *Science* **311**, 1471-4 (Mar 10, 2006)).
