# Supplementary material for: Widespread Protein Aggregation as an Inherent Part of Aging in C. elegans
Source: PLoS Biol. 2010 Aug 10;8(8):e1000450. doi: 10.1371/journal.pbio.1000450 (PMC2919420; doi:10.1371/journal.pbio.1000450)
Supplement: Table S5 — (A) Specific functional categories are over-represented in the age-dependent insoluble protein set. Functional annotation was carried out using the DAVID software. A total of 450 out of 461 age-dependent insoluble proteins were recognized by DAVID and 349 of these fell into one or more significant gene ontology biological process category. EASE score p value: modified Fisher exact p value. (B) Specific functional categories are over-represented in the age-independent insoluble protein set. Functional annotation was carried out using the DAVID software. A total of 243 out of 250 age-independent insoluble proteins were recognized by DAVID and 214 of these fell into one or more significant gene ontology biological process category. EASE score p value: modified Fisher exact p value. (0.02 MB PDF) [file pbio.1000450.s013.pdf]

Table S5A: Specific functional categories are over-represented in the age-dependent insoluble protein set.

Gene ontology term	Number of proteins	% of total	PValue
Embryonic development	248	55%	5.2E-30
Translation	69	15%	4.1E-27
Growth	154	34%	1.8E-17
Protein folding	19	4%	3.9E-07
Determination of adult life span	35	8%	4.4E-07
tRNA aminoacylation	14	3%	2.8E-06
Cytoskeleton organization	23	5%	3.8E-04

Functional annotation was carried out using the DAVID software. A total of 450 out of 461 age-dependent insoluble proteins were recognized by DAVID and 349 of these fell into one or more significant gene ontology biological process category. EASE score P-Value: modified Fisher Exact P-Value.

Table S5B: Specific functional categories are over-represented in the age-independent insoluble protein set.

Gene ontology term	Number of proteins	% of total	PValue
Embryonic development	138	57%	2.4E-24
Translation	33	14%	2E-10
Cellular respiration	14	6%	2.4E-10
Growth	81	33%	2.2E-7
Cofactor metabolic process	16	7%	7.8E-5
Determination of adult life span	19	8%	7.6E-4

Functional annotation was carried out using the DAVID software. A total of 243 out of 250 age-independent insoluble proteins were recognized by DAVID and 214 of these fell into one or more significant gene ontology biological process category. EASE score P-Value: modified Fisher Exact P-Value.
